# Tibetan Medicine for Diabetes Mellitus: Overview of Pharmacological Perspectives

**DOI:** 10.3389/fphar.2021.748500

**Published:** 2021-10-21

**Authors:** Li-Shan Yan, Brian Chi-Yan Cheng, Shuo-Feng Zhang, Gan Luo, Chao Zhang, Qing-Gao Wang, Xiu-Qiong Fu, Yi-Wei Wang, Yi Zhang

**Affiliations:** ^1^ School of Chinese Materia Medica, Beijing University of Chinese Medicine, Beijing, China; ^2^ College of Professional and Continuing Education, Hong Kong Polytechnic University, Hong Kong, China; ^3^ First Affiliated Hospital, Guangxi University of Chinese Medicine, Guangxi, China; ^4^ Centre for Cancer and Inflammation Research, School of Chinese Medicine, Hong Kong Baptist University, Hong Kong, China

**Keywords:** tibetan material medica, diabetes mellitus, insulin resistance, diabetic complications, pharmacology

## Abstract

Diabetes mellitus (DM) and its complications pose a major public health threat which is approaching epidemic proportions globally. Current drug options may not provide good efficacy and even cause serious adverse effects. Seeking safe and effective agents for DM treatment has been an area of intensive interest. As a healing system originating in Tibet, Traditional Tibetan Medicine (TTM) has been widely used by Tibetan people for the prevention and treatment of DM and its complications for hundreds of years. Tibetan Materia Medica (TMM) including the flower of *Edgeworthia gardneri* (Wall.) Meisn., Phyllanthi Fructus, Chebulae Fructus, Huidouba, and Berberidis Cortex are most frequently used and studied. These TMMs possess hypoglycemic, anti-insulin resistant, anti-glycation, lipid lowering, anti-inflammatory, and anti-oxidative effects. The underlying mechanisms of these actions may be related to their α-glucosidase inhibitory, insulin signaling promoting, PPARs-activating, gut microbiota modulation, islet β cell-preserving, and TNF-α signaling suppressive properties. This review presents a comprehensive overview of the mode and mechanisms of action of various active constituents, extracts, preparations, and formulas from TMM. The dynamic beneficial effects of the products prepared from TMM for the management of DM and its complications are summarized. These TMMs are valuable materia medica which have the potential to be developed as safe and effective anti-DM agents.

## Introduction

Diabetes mellitus (DM) is a chronic metabolic disorder with insufficient insulin production or insulin resistance, resulting in a high level of blood glucose (Chatterjee et al., 2017). In the past three decades, DM prevalence has quadrupled globally (Zheng et al., 2018) with 463 million people living with DM worldwide, particularly in low- and middle-income countries, in which 90% are type 2 diabetes mellitus (T2DM) (International Diabetes Federation, 2019). Moreover, DM is a complex metabolic disorder that is accompanied by life-threatening complications, such as diabetic nephropathy diabetic retinopathy and cardiovascular diseases (GBD 2013 Mortality and Causes of Death Collaborators, 2015). According to the World Health Organization (WHO) statistics, approximately 1.5 million deaths were directly attributed to DM in 2019 (Gravina et al., 2021). The increasing trends in the prevalence of disability and premature morbidity impose a considerable economic burden on society (Bommer et al., 2017).

Regardless of classification, the general goal in managing DM includes normalizing blood glucose level and preventing short and long-term complications. Insulin injection is mostly used in patients with type 1 DM (T1DM) (JC, 2012), whereas oral medication is generally prescribed to patients with T2DM, which is usually initiated with metformin as the first-line medicine along with combination therapy including other hypoglycemic agents, such as pioglitazone, sulfonylureas, meglitinides, and α-glucosidase inhibitors (Nathan, 2015). However, current therapies for DM are unlikely to achieve long-term glycemic control. Many classic well-established agents have their own adverse effects and limitations such as abdominal discomfort, gastrointestinal disorders (Dujic et al., 2016), undesired weight gain (Loke et al., 2009) and cardiovascular events (Gilbert and Krum, 2015). New classes of glucose-lowering agents including dipeptidyl peptidase 4 (DPP-4) inhibitors, glucagon-like peptide 1 receptor agonists (GLP-1RAs) and sodium-glucose transport protein 2 (SGLT2) inhibitors provide an opportune moment for DM therapies with a lower risk of side effects and higher therapeutic efficacy. However, these drugs cost far more than the conventional ones and impose a huge financial burden on the patients (Tahrani et al., 2016). Therefore, seeking inexpensive, effective, and safe anti-diabetic agents has always been an area of intensive interest.

As one of the oldest medical systems in the world, Traditional Tibetan Medicine (TTM), also known as Sowa Rigpa in the Tibetan language, is gradually being brought into the limelight with growing attraction to researchers in Asia, and more recently in Europe and North America. With a long history of more than 2,000 years, TTM formed its own medical system that includes the elements of Traditional Chinese Medicine, Indian and Arabic medication (Liu et al., 2009). The theory of TTM is based on the holistic understanding of human health and the natural environment. The functions of the human body were governed by “three humors”, namely, wind, bile, and phlegm (*rlung*, *mkhris pa*, and *bad kan* in Tibetan construct respectively). The imbalance of these three humors is closely relevant to the etiology of various diseases like DM (Finckh, 1984). According to the TTM theory, all herbs consist of five cosmic energies, including space, air, fire, water, and earth (Duo, 1998). Dietary and lifestyle modifications as well as the administration of herbal medicines are often used among Tibetan medical practitioners to regulate the imbalances of these three humors.

Classic herbal pharmacopeia and monographs recorded the use of natural TTM products in managing DM and its complications centuries ago (Fu et al., 2020). Even today, Tibetan Materia Medica (TMM) is still widely used for the treatment of DM in clinics. As the “holistic view” of dynamic balance based on the “three humors” is a unique understanding of DM among Tibetan medical practitioners, treatments for DM and its complications are characteristic advantages of Tibetan medicine. Data from randomized controlled trials (RCTs) conducted in China showed that TMM has potential benefits for the management of various diseases including diabetes and its complications (Namdul et al., 2001; Luo et al., 2015). Notably, accumulated scientific evidences over the past decades indicate that TMM including herbal extracts, Tibetan prescriptions and patent medicine play essential roles in treating DM and its complications (Hao et al., 2018). However, an updated systematic review for TMM in the treatment of DM from pharmacological perspectives is still lacking. In this context, we summarized the traditional uses, active ingredients, and biological/pharmacological activities of TMM concerning its therapeutic value in the management of DM. It is intended to give an overview of the latest scientific knowledge about the impact of TMM on DM and provide a better understanding of the beneficial effects of TMM to DM patients.

## Pathogenesis and Current Management of Diabetes Mellitus

Insulin is a pivotal endocrine peptide hormone secreted by islet β-cell to orchestrate regulatory responses to nutrient sensing. Insulin mediates anabolism in insulin-sensitive tissues, typically including skeletal muscle, liver, and white adipose tissues. These tissues, in turn, feedback information to islet cells about their need for insulin. This feedback loop ensures integration and maintenance of glucose homeostasis (Kahn et al., 2014). DM occurs when there is an imbalance between the demand and production of the insulin. Complete insulin deficiency often caused by an acute destruction of pancreatic beta-cells due to autoimmune disorders, which is the main pathology of T1DM. Moreover, T2DM is characterized by chronic, low-grade inflammation that accompanies by defect in insulin binding to receptors in target tissues (insulin resistance). Genetic mutations, epigenetic changes in relevant genes and environmental factors are important to the development of autoimmune disorders, insulin resistance and β-cell dysfunction in T2DM (Stumvoll et al., 2005; Bluestone et al., 2010). Besides, unhealthy diet, especially increased amounts of dietary fat (saturated fat in particular) and fructose, is one of the key environmental factors that contributes to glucose intolerance and insulin resistance (Hu et al., 2001). DM-related complications are largely ascribed to vascular damage induced by metabolic abnormalities, affecting eyes, feet, kidneys, peripheral, and autonomic nervous systems. Hyperglycemia often causes redox imbalance and inflammation and affects epigenetic pathways (Creager et al., 2003; Forbes and Cooper, 2013). These subsequent factors are also responsible for functional and structural alterations of the vessel wall, which culminates with diabetic vascular complications.

At present, the overall clinical strategy for the prevention and management of DM and its complications aims at achieving effective and sustained glycemic control (Ismail-Beigi, 2012). T2DM can be largely prevented by lifestyle modification in high-risk patients with impaired glucose tolerance. If the lifestyle interventions did not meet the criterion for equivalence for glycemic control, pharmacological therapies are probably required for these T2DM patients. Oral medication is usually initiated with metformin as first-line therapy. If treatment with a single anti-diabetic medication is not satisfactory, a range of combination therapy options with other well-established agents are now available (American Diabetes Association, 2015). When oral medications are unable to control hyperglycemia to recommended levels, insulin injections may be necessary for T2DM patients (Kramer et al., 2013). Long-term administration of conventional oral glucose-lowering drugs, such as sulfonylureas, biguanides, thiazolidinediones, and α-glucosidase inhibitors may cause unpleasant side effects including abdominal discomfort and other gastrointestinal adverse effects (Yang et al., 2014), edema, undesired weight gain and cardiovascular events (Gilbert and Krum, 2015). Compared with these medications, new hypoglycemic agents including GLP-1RAs, DPP-4 inhibitors, and SGLT2 inhibitors have less above adverse effects, but these drugs cost far exceeds than the older agents (e.g. sulfonylureas and meglitinides) (Tahrani et al., 2016).

Although benefit-risk balance for different anti-DM agents varies among patients, early, effective and sustained glycemic control could delay the progression of DM and prevent the hyperglycemia-related complications (Ismail-Beigi et al., 2010). Natural products, which have long been used in traditional systems of medicine for DM, represent an ideal source to explore safe and effective anti-DM agents (Xu et al., 2018; Semwal et al., 2021). There are numerous studies investigated the anti-DM property of TTM and the common therapeutic targets including α-glucosidase, PPARs (peroxisome proliferator-activated receptors), insulin signaling pathways, β-cell function and gut microbiota (Pourcet et al., 2006; Kumar et al., 2011; Li B. Y. et al., 2019; Li J. et al., 2019; Semwal et al., 2021). Meanwhile, suppression of pro-inflammatory, pro-fibrogenic and angiogenic cytokines, such as vascular endothelial growth factor (VEGF) and transforming growth factor β (TGF-β) is referred as a potent therapeutic approach for DM and its complications (Joshi et al., 2019; Lu et al., 2019). Thus, the multi-target characteristics of TTM may be advantageous over single-target drugs in the treatment of complex metabolic diseases like DM.

## Tibetan Medicine Theory of Diabetes Mellitus

According to *Four Treatises* or *Four Tantras* (Si Bu Yi Dian), one of the most classic literatures of TTM, DM is categorized as “gcin snyi sa khu” disease in traditional Tibetan medical theory (Dhangombo, 1983). The word “gcin snyi” in Tibetan refers to frequent urination and “sa khu” means consumption and impurity, suggesting that the essential feature of DM in a TTM theory is excess urination and emaciation thereby gradually wearing the body down. This understanding of DM is consistent with the modern medicine theory which elucidates the pathological syndromes of DM. In Tibetan theory of medicine, the fundamental point of view about the physiology and pathology of DM is built on the notions of three humors. Since TTM views health as a state of balance, the etiology of DM is divided into internal and external causes based on the imbalance of three humors. The internal causes mainly refer to mental confusions inclusive of greed, anger and ignorance that may lead to dysfunction of *rlung*, *mkhris pa*, and *bad kan*. The external causes include improper diet, damp environment and other unhealthy lifestyles, consequently giving rise to the overflow of *bad kan* and fat through the body. Accordingly, there are 3 subtypes of DM: *rlung* type, *mkhris pa* type, and *bad kan* type (Zhong, 2014). As discussed in *Four Tantras*, *bad kan* type is identified as over-nutrition followed by hyperglycemia and obesity. *Mkhris pa* type shows weakened ability of glucose uptake and utilization and thus the body breaks down stored fat for energy which leads to rapid emaciation and ketonuria. *Rlung* type appears with diabetic complications such as palpitation, insomnia and glaucoma. Therefore, a comprehensive Tibetan diagnosis helps to identify the condition of disease progression (Dakpa and Dodson-Lavelle, 2009) and with the involvement of personalized clinical interventions, TTM exhibits great therapeutic value in DM and its complications.

Tibetan therapeutic prescriptions often begin with dietary and lifestyle modifications followed by oral administration of herbal medicines to restore the balance of the three humors. TTM physicians also recommend patients engage in self-regulatory practices (i.e., yoga and meditation) as a strategic approach for treating the body and mood in tandem. Natural Tibetan medicine and patent medicine are most widely used for the treatment of DM and its complications in Western China, which have drawn considerable attention among researchers to identify the active constituents and explore the underlying mechanisms of their anti-DM action.

## Commonly Used Tibetan Materia Medica for Diabetes Mellitus Treatment

In recent years, TMM has increasingly been accepted by more and more people worldwide, especially for the management of DM, and the researches for the anti-diabetic effects of TMM become increasingly popular (Song et al., 2016). Here, we performed a large-scale text mining of PubMed and China National Knowledge Infrastructure (CNKI). We extracted the promising anti-diabetic TMM from the English and Chinese scientific literatures (from Jan. 1st^,^ 2000 to Jun. 15th^,^ 2021) with the keywords “Tibetan medicine” and “diabetes”. Moreover, Google Patents and Chinese patent database (http://pss-system.cnipa.gov.cn) were also used to obtain the full spectrum of applications of TMM in anti-diabetic prescription research. Relevance to DM was calculated by the ratio of the number of DM-TMM-related papers to the volume of papers about one single TMM. A *p* value was calculated to determine the improbability of co-occurrences of each TMM and DM occur by chance:

$$p=1-\sum_{i=0}^{k}f\left(i\right)=1-\sum_{i=0}^{k}\frac{\left(\begin{array}{c} K\\ i \end{array}\right)\left(\begin{array}{c} N-K\\ n-i \end{array}\right)}{\left(\begin{array}{c} N\\ n \end{array}\right)},$$ (1)

where *N* is the total number of journal articles in PubMed and CNKI (62,722 404 articles from January 1st, 2000 to June 15th^,^ 2021, 16,497 978 in PubMed and 46,224 426 in CNKI, respectively), *K* is the number of papers associate with DM in the meanwhile (798,867‬ articles in total, 413,090 in PubMed and 385,777 in CNKI, respectively), *n* is the number of papers of TMM, *k* is the number of papers about the anti-diabetic effects of TMM. The value of *N, K, n,* and *k* was obtained from PubMed and CNKI. *p*-value indicates the significance of relevance between each TMM and DM (when *p*-value < 0.01 means significant) (Sun et al., 2013).

**FIGURE 1:**
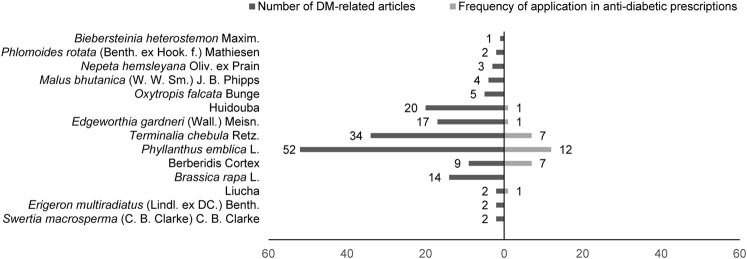
Reported Tibetan medicinal plants/medicinal materials that are associated with DM treatment.

Among 195 collected DM-TMM-related research articles, 17 species of Tibetan medicinal plants distributed in 11 families were reported to be associated with the treatment of DM (Figure 1). Among these TMM, Chebulae Fructus [fruits of *Terminalia chebula* Retz. (Family: *Combretaceae* R. Br.)], Phyllanthi Fructus [fruits of *Phyllanthus emblica* L. (Family: *Phyllanthaceae* Martinov)], the flower of *Edgeworthia gardneri* (Wall.) Meisn. (Family: *Thymelaeaceae* Juss.), Huidouba and Turnip [roots of *Brassica rapa* L. (Family: *Brassicaceae* Burnett)] are the top well studied (Table 1).

**TABLE 1:** Correlations between TMM with DM.

TMM	Volume of articles	
	Total	Relevant to DM (ratio; *p*-value)
The flower of *Edgeworthia gardneri* (Wall.) Meisn	34	17 (3 are reviews) (50.00%; *p* < 0.001)
Phyllanthi Fructus	1,254	52 (5 are reviews) (4.15%; *p* < 0.001)
Chebulae Fructus	1,261	34 (4 are reviews) (2.70%; *p* < 0.001)
Huidouba	39	20 (including one review) (51.28%; *p* < 0.001)
Turnip (root of *Brassica rapa* L.)	2,184	14 (including one review) (0.64%; *p* = 0.997)

The results showed that the flower of *E. gardneri* and Huidouba were significantly correlated with DM, suggesting that anti-DM may be one of the major therapeutic effects for these two TMMs. Studies on the fruits of *T. chebula* Retz. and *P. emblica* L. emphasized their multiple pharmacodynamic activities rather than their anti-diabetic properties. Meanwhile, Chebulae Fructus and Phyllanthi Fructus were the most commonly used TMM in prescriptions for DM treatment among registered pharmaceutical patents and formulas under scientific investigation (Table 2), such as Padma 28 and Triphala. Besides, there was an intense research interest on Turnip, one of the oldest cultivated vegetables in the world. Turnip possesses diverse health benefits especially for its anti-DM action (Paul et al., 2019). In the following sections, major findings on the therapeutic value of representative TMM in DM and its complications are reviewed.

**TABLE 2:** TMM used in anti-DM prescriptions and registered patents.

TMM	Patent publication number	Herbal formula
Phyllanthi Fructus	CN-1899565-A	Siwei Jianghuang Decoction Powder; Triphala; Eighteen-Flavor *Myrobalan* Diuretic pills; Jikan Mingmu Drops; Tang-Kang-Fu-San
	CN-103948666-A	
	CN-103961551-A	
	CN-103961392-A	
	CN-105106644-A	
	CN-109331147-A	
	CN-108404063-A	
Chebulae Fructus	CN-105106644-A	Padma 28; Triphala; Eighteen-Flavor *Myrobalan* Diuretic pills; Jikan Mingmu Drops
	CN-108653682-A	
	CN-108404063-A	
The flower of *Edgeworthia gardneri* (Wall.) Meisn	CN-106620085-A (LLKL)	
Berberidis Cortex	CN-1899565-A	Siwei Jianghuang Decoction Powder; Eighteen-Flavor *Myrobalan* Diuretic pills; Tang-Kang-Fu-San; Jikan Mingmu Drops
	CN-103961551-A	
	CN-108404063-A	
Liucha [young leaves and shoots of *Sibiraea laevigata* (L.) Maxim. and *Sibiraea angustata* (Rehder) Hand.-Mazz.]	CN-106620085-A	LLKL
Huidouba	—	Compound HDB, (Zhao et al., 2011)

### *Edgeworthia gardneri* (Wall.) Meisn

The flower of *Edgeworthia gardneri* (Wall.) Meisn. (Lv-luo-hua in Chinese) has been commonly consumed as a healthy beverage in Tibet to ameliorate metabolic disorders. It has been reported that the extract of the flower of *E. gardneri* has a wide array of pharmacological activities, such as anti-hyperglycemia, anti-adipogenesis, α-glucosidase inhibition, and anti-oxidation (Li et al., 2020). The main active components of the flower of *E. gardneri* have been identified including coumarins, flavonoids, polysaccharides, and polyphenols (Xu et al., 2012). Recent studies focused more on the biological activities of crude extracts of *E. gardneri*, such as water extract, n-hexane extract, ethyl acetate extract and petroleum ether extract*.* These extracts could lower blood glucose levels, improve glucose metabolism and lipid disorder in various diabetic animal models (Bao et al., 2019; Li et al., 2020).

The hypoglycemic effect of *E. gardneri* may be related to its α-glucosidase inhibitory action. The ethyl acetate extract of *E. gardneri* was found to possess potent inhibitory effect on α-glucosidase (Geng et al., 2013). Moreover, many reports indicated that some compounds isolated from *E. gardneri* exhibited anti-DM activity both *in vitro* and *in vivo*. Tiliroside, identified as the major compound of the phenolic fraction from EtOAc extract of *E. gardneri*, showed a strong α-glucosidase inhibitory effect with an IC_50_ value of 202 μg/ml (Ma et al., 2015). A significant reduction in the postprandial blood glucose levels of normal and STZ-induced diabetic mice was found after oral administration of tiliroside at a dose of 300 mg/kg. Tiliroside also decreased the fasting blood glucose (FBG) levels in diabetic mice, showing the potential to be an attractive anti-hyperglycemic agent for DM treatment. Daphnoretin, one of the coumarins isolated from the EtOAc fraction, displayed suppressive effect on the activities of α-amylase and α-glucosidase with IC_50_ of 90 and 86 μg/ml, respectively (Zhao et al., 2015). Notably, compared with classic α-glucosidase inhibitor acarbose with IC_50_ of 2.8 μg/ml on the anti-α-amylase activity, daphnoretin seems to have a milder α-amylase suppressive effect, which may improve prolonged inhibition of starch hydrolysis and thus might be expected to cause fewer gastrointestinal adverse effects (Dehghankooshkghazi and Mathers, 2004).

Additionally, *E. gardneri* has been shown to promote PPARs activities which play crucial roles in regulating glucose and lipid metabolism. It has been reported that n-hexane, ethyl acetate and n-butanol extracts of *E. gardneri* could activate PPARγ and PPARβ *in* cell models (Gao et al., 2015; Lin and Xia, 2016). Umbelliferone and pentadecanoic acid separated from the secondary fractions of EtOAc extract of *E. gardneri* were further identified as potential dual agonists for PPARγ/β (Gao et al., 2015). The activation of PPARs by *E. gardneri* may be partially attributed to its fatty acid composition, such as pentadecanoic acid (Han et al., 2009), which could bind to PPARs as endogenous ligand and then activates it (Wang L. M. et al., 2014). Daphnoretin, a dicoumarol component from *E. gardneri*, presented to be a PPARα/β/γ pan-agonist both in insulin-resistant hepatocyte and adipocyte models (Li et al., 2018; Nan et al., 2019), which may have the potential to improve metabolic disorder (Kaplan et al., 2001).

Studies also found that *E. gardneri* improved insulin resistance in diabetic models. Our recent study showed that the water extract of the flower of *E. gardneri* (WEE) ameliorated palmitate-induced insulin resistance through regulating IRS1/GSK3β/FoxO1 signaling pathway in HepG2 hepatocytes (Zhang Y. et al., 2020). Additionally, n-hexane extract of *E. gardneri* (EGH) improved insulin resistance in skeletal muscle via elevating GLUT4 expression both *in vivo* and *in vitro* (Meng et al., 2019). This study further indicated that fraction 1 isolated by gel chromatography from EGH could attenuate PA-induced insulin resistance in C2C12 cells *via* modulating IR/AMPK/GLUT4 signaling pathway (Meng et al., 2019). In addition, gut microbial imbalance affects carbohydrate, lipid, and amino acid metabolism and plays an important role in the pathogenesis of insulin resistance (Cani and Delzenne, 2009). WEE could reduce homeostasis model assessment of insulin resistance index in diabetic mice (Zhang et al., 2019). High-throughput 16S rRNA-Seq analysis further showed that WEE regulated gut bacterial phylotypes to restore the balanced gut microbiota. WEE also increased the content of short chain fatty acids secreted by the gut microbiota to improve gut barrier functions and ameliorate insulin resistance (Zhang et al., 2019).

Besides, quercetin (10 μmol/L) extracted from *E. gardneri* markedly enhanced insulin secretion in the presence of glucose (8.3 mmol/L) in MIN-6 cell (Zhuang et al., 2018). In the case of glucose-induced insulin secretion, exocytosis of insulin granules is initiated by intracellular calcium influx through plasmalemmal voltage-dependent calcium channels (VDCCs) (Rorsman and Ashcroft, 2018). Then extracellular-signal-regulated kinase (ERK) phosphorylation driven by VDCC-induced cAMP accumulation is rapidly activated on the plasma membrane upon glucose stimulation (Pratt et al., 2016). Moreover, calcium channel inhibitor nifedipine and ERK1/2 inhibitor AZD8330 inhibited quercetin-induced insulin secretion, indicating that quercetin enhanced insulin secretion via calcium and ERK1/2 signaling pathways (Zhuang et al., 2018). Meanwhile, the decline of mitochondrial membrane potential is an early hallmark of apoptosis (Barbu et al., 2002). Quercetin treatment inhibited palmitic acid induced cell apoptosis by suppressing the activation of caspase-3, -9, -12. It also increased the ratio of Bcl-2/BAX and attenuated the impaired mitochondrial membrane in MIN-6 cells (Zhuang et al., 2018).

### *Phyllanthus emblica* L

*Phyllanthus emblica* L., whose fruits are also known as Indian gooseberry and “skyu-ru-ra” in Tibetan, is a widely used medicinal plant in Indian and Tibetan folk medicine. The fruits of *P. emblica* have been intensively studied and have potential to manage various diseases like DM. Clinical study indicated that administration of *P. emblica* fruit powder for 3 weeks could significantly lower blood glucose and lipids in diabetic patients (Akhtar et al., 2011). Moreover, after treatment with water extract of Phyllanthi Fructus, the improvement of endothelial function, oxidative stress and inflammation were observed in T2DM patients (Usharani et al., 2013). Pre-clinical research illustrated that Phyllanthi Fructus attenuated hyperglycemia as a potent α-glucosidase inhibitor and free radical scavenger *in vitro* (Nampoothiri et al., 2011; Majeed et al., 2020). Phyllanthi Fructus aqueous extract also elevated the expression of PPARγ and adiponectin in adipose tissue of high fat diet induced obese rats, representing a potential target of PPARγ activation (Xi et al., 2009). Phyllanthi Fructus extract was also reported to alleviate insulin resistance *via* increasing the expression of PI3K, Akt, and GLUT4 in skeletal muscle of STZ-induced diabetic rats (Dong et al., 2009; Hu, 2012).

Gallic acid (GA), one of the bioactive polyphenolic components isolated from Phyllanthi Fructus, activated PPARγ and C/EBP in an Akt-dependent manner and upregulated the expression of GLUT4 thereby promoting glucose uptake in 3T3-L1 adipocytes (Variya et al., 2020). GA also improved metabolic parameters in *db/db* mice and fructose-fed SD rats (Variya et al., 2020). GA could down-regulate the expression of thioredoxin-interacting protein (TXNIP) to ameliorate oxidative stress and endoplasmic reticulum stress, thereby promoting glucose uptake and alleviating pancreatic β-cell glucose toxicity. GA also suppressed the expression of NOD-, LRR-, and pyrin domain-containing protein 3 (NLRP3) and formation of NLRP3 inflammasome and markedly ameliorated glucotoxicity-induced apoptosis of β-cell *in vivo* and *in vitro* (Zuo et al., 2018). In addition to GA, its dimeric derivative ellagic acid (EA) displays a remarkable anti-diabetic activity by protecting islet β-cells. EA improved glucose-stimulated insulin secretion from isolated islets and glucose tolerance in STZ-induced T2DM rats. It also decreased thiobarbituric acid-reactive substances (TBARS, a marker of lipid peroxidation) while increased plasma total antioxidants and liver GSH, which may contribute to the improved β-cell mass and function (Fatima et al., 2017).

In addition, Phyllanthi Fructus exhibits strong protective effect on DM-related complications. The enriched tannoids isolated from *P. emblica* obviously inhibited both rat lens and human aldose reductase (AR) with IC_50_ of 6 and 10 μg/ml respectively (Suryanarayana et al., 2004). The tannoids fraction also prevented sugar-induced AR activation and the osmotic changes in cultured rat lens organs, suggesting its therapeutic potential for cataract. Further study demonstrated that cataract progression was delayed in tannoids-treated diabetic rats (Suryanarayana et al., 2007). This may be contributed to its inhibitory effect on AR activity and sorbitol accumulation by Emblica tannoids. Furthermore, 1-O-galloyl-β-D-glucose (β-glucogallin), one of the metabolites and major biosynthetic precursors of tannoids, is presented as a selective inhibitor of human AR *in vitro* (Puppala et al., 2012). β-glucogallin also repressed sorbitol accumulation in AKR1B1 transgenic mouse lenses under high glucose exposure (Puppala et al., 2012). Besides β-glucogallin, another metabolite of ellagitannin, urolithin A (UroA) could prevent vascular smooth muscle cell proliferation induced by hyperglycemia *via* regulating Akt/Wnt/β-catenin signaling pathway (Zhou et al., 2018). Phyllanthi Fructus could inhibit glycation reaction by reducing glycosylated protein level *in vitro* (Rao et al., 2005; Nampoothiri et al., 2011). *P. emblica* was also reported to attenuate diabetic neuropathy and relieve neuropathic pain, suggesting its anti-oxidative and anti-inflammatory properties (Kumar et al., 2009; Tiwari et al., 2011).

### *Terminalia chebula* Retz

*Terminalia chebula* Retz., the fruit of which is commonly known as Chebulic Myrobalan or Haritaki, is one of the major medicinal plants in Tibetan and Ayurvedic systems of medicine (Sharma et al., 2019). In Tibet, Haritaki is called “a-ru-ra” and recognized as “man-mchog-rgyal-lo” which means “king of medicines” (Meher et al., 2018). Haritaki is often used in traditional medicine for its wide spectrum of pharmacological activities, such as anti-inflammation, anti-oxidation, anti-DM, and hepato-protection (Gupta, 2012; Silawat and Gupta, 2013). The health benefits may be associated with the presence of various phytochemicals such as polyphenols, anthocyanins, terpenes, flavonoids, alkaloids, and glycosides (Muhammad et al., 2012). Network pharmacology research revealed that 4 potential targeted compounds isolated from *T. chebula* extracts, including EA, luteolin, chebulic acid and quercetin have appropriate oral bioavailability and drug-like properties (Wang D. Y. et al., 2014). It has been reported that EA, luteolin and quercetin inhibited the activities of α-glucosidase *in vitro* (Tabopda et al., 2008; Li et al., 2009; Yan et al., 2014). Three active ellagitannins including chebulanin, chebulagic acid and chebulinic acid isolated from the aqueous methanolic extract of dried fruit of *T. chebula* possess potent rat intestinal maltase inhibitory activity (Gao et al., 2007). Chebulagic acid was further proved to be a reversible and non-competitive inhibitor of maltase (Gao et al., 2008). It has also been reported that chebulagic acid significantly reduced post-administration blood glucose level of SD maltose-loaded male rats (Huang et al., 2012). The above findings suggested that Haritaki may delay carbohydrate digestion by inhibiting α-glucosidase activity with maltase as the main substrate.

Moreover, three gallotannins isolated from *T. chebula*, including 2,3,6-tri-O-galloyl-β-D-glucose, 1,2,3,6-tetra-O-galloyl-β-D-glucose and PGG were found to enhance insulin-stimulated glucose uptake through elevating PPARα/γ expression in HepG2 cells. Meanwhile, these compounds did not promote adipogenesis in 3T3-L1 cells, suggesting that these compounds increased cellular glucose uptake through activation of PPARs thereby accelerating glucose consumption (Yang et al., 2013). *T. chebula* methanolic extract containing 2.7% chebulic acid (CA) and EtOAc-soluble portion of ethanolic extract of *T. chebula* fruit containing 29.4% CA was reported to present preventive effects against the aggregation of advanced glycation end products (AGEs) (Kim et al., 2011; Lee et al., 2011). A further *in vitro* study illustrated that CA isolated from Terminalia chebula inhibited glycolaldehyde-glycated bovine serum albumin induced collagen cross-link formation and broke existent cross-links *via* its chelating and antioxidant activities (Lee et al., 2014). CA prevented glyceraldehyde-related AGEs (glycer-AGEs)-induced transendothelial electrical resistance reduction on Human umbilical vein endothelial cell (HUVEC), indicating enhanced endothelial barrier function (Lee et al., 2010). Increased adhesion of HUVEC with human monocytic THP-1 cells induced by glycer-AGEs was remarkably suppressed after CA treatment (Lee et al., 2010). In addition, in silico experiments showed that CA could bind with TGF-β receptor thus inhibiting the downstream signaling and tissue fibrosis (Shanmuganathan and Angayarkanni, 2019). The above studies suggest that *T. chebula* especially its bioactive component chebulic acid can act as agents for the treatment of DM and its complications.

### Huidouba

Huidouba (HDB), the strip-shaped or bag-shaped cobwebs of spiders of the genus *Atypus* which lived on tea plants in Mount Emei, has long been used for DM management in Sichuan and Tibet provinces. As a folk medicine and functional food, HDB could improve diabetic symptoms and prevent diabetic complications with a reputation as “the enemy of DM” in the local area (Yang and Kang, 2017). HDB contains various compositions including proteins, polysaccharides, flavonoids, saponins and alkaloids among which polysaccharides are the main effective components (Wu et al., 2009). Several studies indicated that polysaccharides purified from HDB exhibited potential suppressive effect on α-glucosidase activity *in vitro* and *in vivo*. Crude polysaccharides from Huidouba (CHDBP) as well as purified HDBP-1 and HDBP-2 substantially inhibited α-glucosidase activities, with IC_50_ values of 0.019, 0.039, and 0.013 mg/ml, respectively. They were more potent than the positive control namely acarbose (IC_50_ = 0.097 mg/ml). Furthermore, heteropolysaccharide HDBP-2 with lower molecular weight (9.703 kDa) performed better in antioxidant capacity, α-glucosidase inhibitory and hypoglycemic activities (Yan et al., 2014; Chen et al., 2018). This may be attributed to their easier access to the cellular interior with fewer difficulties passing through structural barriers as for relatively smaller molecules. In addition, these polysaccharides decreased the serum triglyceride (TG), total cholesterol (TC), and low-density lipoprotein cholesterol (LDL-C) levels and significantly improved oral glucose tolerance (OGT) in T2DM KKAy mice, indicating a potential to ameliorate T2DM symptoms. Apart from polysaccharides, protein components of HDB also showed potential for meliorating DM. In an alloxan-induced diabetic mouse model, among polysaccharide, protein and alkaloid extracts, HDB proteins showed profound hypoglycemic activity with strong inhibitory effect on α-glucosidase while the combined extract was superior to all these fractions (Wu et al., 2013). The coordination of multiple components from the combined extract is likely to enhance its hypoglycemic performance. High throughput screening assay also indicated that ethyl acetate extract and n-hexane extract of HBD displayed remarkable activation on PPARγ and PPARβ (Lin and Xia, 2016). The EtOAc and n-hexane extracts mainly contain volatile oil, fatty acids, terpenoids and some alkaloids with low polarity, which may improve their interaction with the binding site of PPARs.

Meanwhile, studies found that HDB could improve renal function in diabetic models. In the mouse model of diabetic nephropathy, HDB ethanol extract, water extract and polysaccharides administration decreased the glucose, creatinine, urea nitrogen, urine protein, glycosylated serum protein and AGEs in serum (Zhou et al., 2018). HDB extracts also alleviated the glomerular basement membrane (GBM) thickening, mesangial matrix expansion, renal tubular and epithelial cells degeneration. The extracts alleviated the renal damage *via* inhibition of the expression of fibrosis and inflammation markers including fibronectin (FN), intercellular adhesion factor-1 (ICAM-1) and TGF-β1. In particular, HDB aqueous extract markedly reduced malondialdehyde (MDA) in the kidney and downregulated the expression of Nox4 predominantly located in the glomerular tissue while upregulated nephrin and WT1 expression in DN rat, implying a potent antioxidant ability to ameliorate renal injury (Yang et al., 2020). HDB proteins also displayed a significant suppressive effect on aldose reductase, thereby attenuating oxidative stress (Wu et al., 2013).

### Berberidis Cortex

Plants belonging to the genus *Berberis* (Berberidaceae) are widely distributed in subtropical and temperate regions with nearly 550 species (Mokhberdezfuli et al., 2014). Berberidis Cortex, the dried root or stem bark of *Berberis kansuensis* Schneid. and its relative plants, is one of the most commonly used TMMs and the key ingredients of a variety of prescriptions for the treatment of chronic metabolic diseases. *B. vernae* Schneid. and *B. dictyophylla* Franch. are widely used in clinics. Extracts and compounds obtained from *Berberis* species, especially Berberine alkaloid (BBR), showed potential abilities to treat DM and other metabolic diseases (Belwal et al., 2020).

Recent pharmacological studies showed that the levels of FBG, glycated serum protein, insulin, insulin resistance index, TNF-α and IL-6 were decreased after 30-days treatment with the water extract of dried stem bark of *B. kansuensis* in T2DM rats (Du et al., 2020; Xu et al., 2021). ^1^H NMR-based metabolomics indicated that among 28 metabolites identified in rat serum, 8 of them including isoleucine, acetoacetate, betaine, valine, glucose, glutamate, N-acetyl glycoproteins, and LDL/VLDL were significantly reversed after *B. kansuensis* treatment. Further analysis of metabolic pathways revealed that the anti-diabetic effect of *B. kansuensis* might be related to the modulation of glycolysis and gluconeogenesis, citric acid cycle, lipid metabolism, amino acid metabolism, and choline metabolism. Meanwhile, *B. kansuensis* extract regulated gut microbiota composition in T2DM rats (Xu et al., 2021). Of note, *B. kansuensis* extract did not improve insulin resistance in pseudo-germ-free T2DM rats, suggesting that the anti-diabetic effect of *B. kansuensis* may be closely associated with its modulation of gut microbiota.

Numerous studies revealed that BBR posses insulin-sensitizing effects. BBR concentration-dependently increased the expression of insulin receptor (IR) expression in cultured human liver cells and L6 rat skeletal muscle cells through regulating protein kinase C (PKC)-dependent signaling (Kong et al., 2009). BBR prevented mitochondrial dysfunction and increased mitochondrial biogenesis and AMPK activation in C2C12 myoblasts (Gomes et al., 2012). However, this improvement was largely inhibited by knockdown of sirtuin 1 (SIRT1), suggesting that BBR is likely to stimulate mitochondrial biogenesis *via* a SIRT1-mediated mechanism which protects muscle from diet-induced insulin resistance. BBR also inhibited the phosphatase activity of protein tyrosine phosphatase 1B (PTP1B) (Chen et al., 2010), which catalyzed IR dephosphorylation in adipocytes but could be repressed by SIRT1 (Shi et al., 2004; Sun et al., 2007). Therefore, the insulin-sensitizing effect of BBR is probably mediated by SIRT1-dependent inhibition of PTP1B activity. Moreover, BBR improved insulin resistance through alleviation of gut microbiota dysbiosis in *db/db* mice (Liu et al., 2018). These findings indicate that BBR has potential ability to ameliorate insulin resistance.

In addition, prescriptions prepared from Berberidis Cortex are commonly used in the management of DM in Tibet. Tibetan medicine formulas such as Tang-Kang-Fu-San, Siwei Jianghuang Decoction and Jikan Mingmu Drops, exhibited potential anti-diabetic effects (Duan et al., 2017b; Lai et al., 2018; Ai et al., 2019). Tang-Kang-Fu-San was reported to promote Akt and AMPK activities, thereby increasing GLUT4 translocation to the plasma in skeletal muscles of *db/db* mice to improve insulin resistance (Duan et al., 2017a). Siwei Jianghuang Decoction Powder (SWJH) documented originally in *Four Tantras*, is often used in kidney disease treatment. SWJH extract could alleviate hyperglycemia, renal fibrosis, and GBM thickness in *db/db* mice (Lai et al., 2018). Furthermore, hypoxia inducible factor-1α (HIF-1α) is a transcription factor induced by hypoxia reaction and could activate the expression of target genes such as VEGF to exacerbate fibrosis and inflammation (Mayer, 2011). SWJH and its bioactive component BBR could inhibit the overexpression of HIF-1α, TGF-β1, and VEGF in *db/db* mice thus ameliorate the renal damage (Lai et al., 2018). Jikan Mingmu Drops (JMD), containing six herbs including Berberidis Cortex, is currently used to treat diabetic induced dry eye syndrome (DES). JMD treatment markedly increased the tear volume, decreased the corneal fluorescein staining score, restored the morphology and structure of conjunctival epithelial cells in benzalkonium chloride induced DES model of *db/db* mice (Ai et al., 2019). This may be associated with its suppressive effect on the production of inflammatory mediators.

### Triphala

Triphala (THL), also known as Zhe-Bu-Song decoction, is composed of the fruits of three Tibetan herbs, including Phyllanthi Fructus, Chebulae Fructus, and Fructus Terminaliae Billericae [fruits of *Terminalia bellirica* (Gaertn.) Roxb.]. THL is one of the most common basic prescriptions accessible in both Ayurveda and Tibetan medicine for balancing and revivifying the three humors (Peterson et al., 2017). A recent clinical study showed that blood glucose and lipid levels were significantly reduced in T2DM patients after 12-months treatment of THL (Singh et al., 2015). Meanwhile, the activity of antioxidant enzymes was notably up-regulated and DNA damage was also attenuated in THL-administered patients. Long-term administration of THL did not show obvious toxicity, which illustrated that THL was a promising therapeutic candidate as an alternative anti-diabetic drug with high safety performance.

Incretins such as GLP-1 and glucose-dependent insulinotropic polypeptide (GIP) are released from the gut, which potentiate insulin release in a glucose-dependent manner (Baggio and Drucker, 2007) and modulate β-cell energetics in intact islets of Langerhans (Hodson et al., 2014). Recent study showed that the size of islet and the number of β-cell granules stained in deep purple was notably enhanced after 6-weeks of THL administration in STZ-induced diabetic rats, indicating a preservative effect of THL on islet β-cell. THL up-regulated the components of incretin/cAMP signaling pathway including GIP, GLP-1, GLP-1R, and cAMP and promoted the phosphorylation of PKA (Zhang Y. J. et al., 2020). It also down-regulated the expression of TXNIP, a vital modulator participating in pancreatic β-cell bioactivity and an indicator of oxidative stress involved in DM progression (Schulze et al., 2004; Chen et al., 2008). This study suggested that THL may enhance the activity of incretin/cAMP signal pathway to affect the proliferation and apoptosis of islet β cells. In addition, supplementation of THL for 45 days obviously reduced the blood glucose levels in non-insulin-dependent diabetic patients (Rajan and Antony, 2008). It has been reported that the hypoglycemic effect by THL may be related to its pancreatic glycolytic enzyme suppressive effects and anti-glycation potential (Ganeshpurkar et al., 2015).

In addition, alcoholic extract of THL (AlE) along with its bioactive principles CA, chebulinic acid (CI) and GA displayed anti-inflammatory and anti-angiogenic properties in diabetic cell models (Shanmuganathan and Angayarkanni, 2018). In DM, hyperglycemia, hypertension and dyslipidemia induce oxidative stress, nuclear factor-κB (NF-κB) activation, dysregulation of NOS, and formation of AGEs, leading to endothelium dysfunction and the resultant complications (Hink et al., 2001; Giacco and Brownlee, 2010). Chronic inflammation is considered to be one of the characteristic features seen at sites of diabetic complications (Feng et al., 2005). Elevated circulating pro-inflammatory cytokines and chemokines have been shown to influence glial cell and neuron behavior, contributing to the pathological processes relevant to both diabetic neuropathy and retinopathy (Doupis et al., 2009; Mesquida et al., 2019). Treatment with CA/CI/GA and AlE markedly suppressed TNF-α-induced pro-inflammatory and pro-angiogenic changes in chorioretinal microvascular endothelial cells (RF/6A) (Shanmuganathan and Angayarkanni, 2018). This was mediated by remarkable inhibition of p38, ERK, and NF-κB phosphorylation and subsequent production of cytokines. In silico studies further revealed that CA, CI, and GA have the potential to bind TNFα-receptor-1 thereby blocking TNF-α signaling transduction. Collectively, as a polyherbal formula, THL exerts various pharmacological properties for protection against DM, which highlights its clinical effects on treatment for DM and its complications.

### Padma 28

Padma are a series of herbal patent medicines and food supplements which are manufactured as commercial products on a large scale and consumed globally. Padma 28, based on the well-known 28th formula of a traditional Tibetan formula collection, is a Swiss herbal remedy that consists of over 22 herbal drugs. It has been reported that Padma 28 could improve circulatory disorders such as intermittent claudication in clinical trials (Stewart et al., 2016). A systematic review interpreted that indications of efficacy were also found in other vascular diseases, inflammatory conditions and lipid metabolism disturbances (Vennos et al., 2013), which suggested that Padma 28 may have the ability to repair metabolic and immune functions.

This multi-herbal preparation is rich in secondary plant substances including essential oils, flavonoids, and tannins (Samochowiec and Wieland, 1983). Herbal flavonoids isolated from Padma 28 was investigated to explore the protective effect on the autoimmune disorder in non-obese diabetic mice (Weiss et al., 2011). The destruction of β-cells in autoimmune DM involves an imbalanced Th1/Th2 polarization and Th1/Th2 cytokines (Rabinovitch and Suarez-Pinzon, 1998). The aqueous extract of Padma 28 containing abundant flavonoids was found to suppress the development of autoimmune DM in female NOD mice (Weiss et al., 2011). The levels of Th2 cytokines IL-10 and IL-6 were increased whereas Th1 cell-derived IL-12 was decreased after intraperitoneal administration of aqueous Padma 28 extract. In the meantime, the proportion of CD8^+^ type 1 cytotoxic T cells, which are known to be a destructor of islet β-cells (Pinkse et al., 2005), was reduced by Padma 28 extract treatment. This suggested that Padma 28 water extract regulated Th1/Th2 balance, which may have potential ability to hinder the development and progression of T1DM (Weiss et al., 2011). Additionally, 70% ethanolic extracts of Padma 28 and Padma Circosan significantly inhibited the formation of AGEs and advanced oxidation protein products to a similar extent as the active controls aminoguanidine *in vitro* (Grzebyk and Piwowar, 2014).

## Conclusion and Implications

**FIGURE 2:**
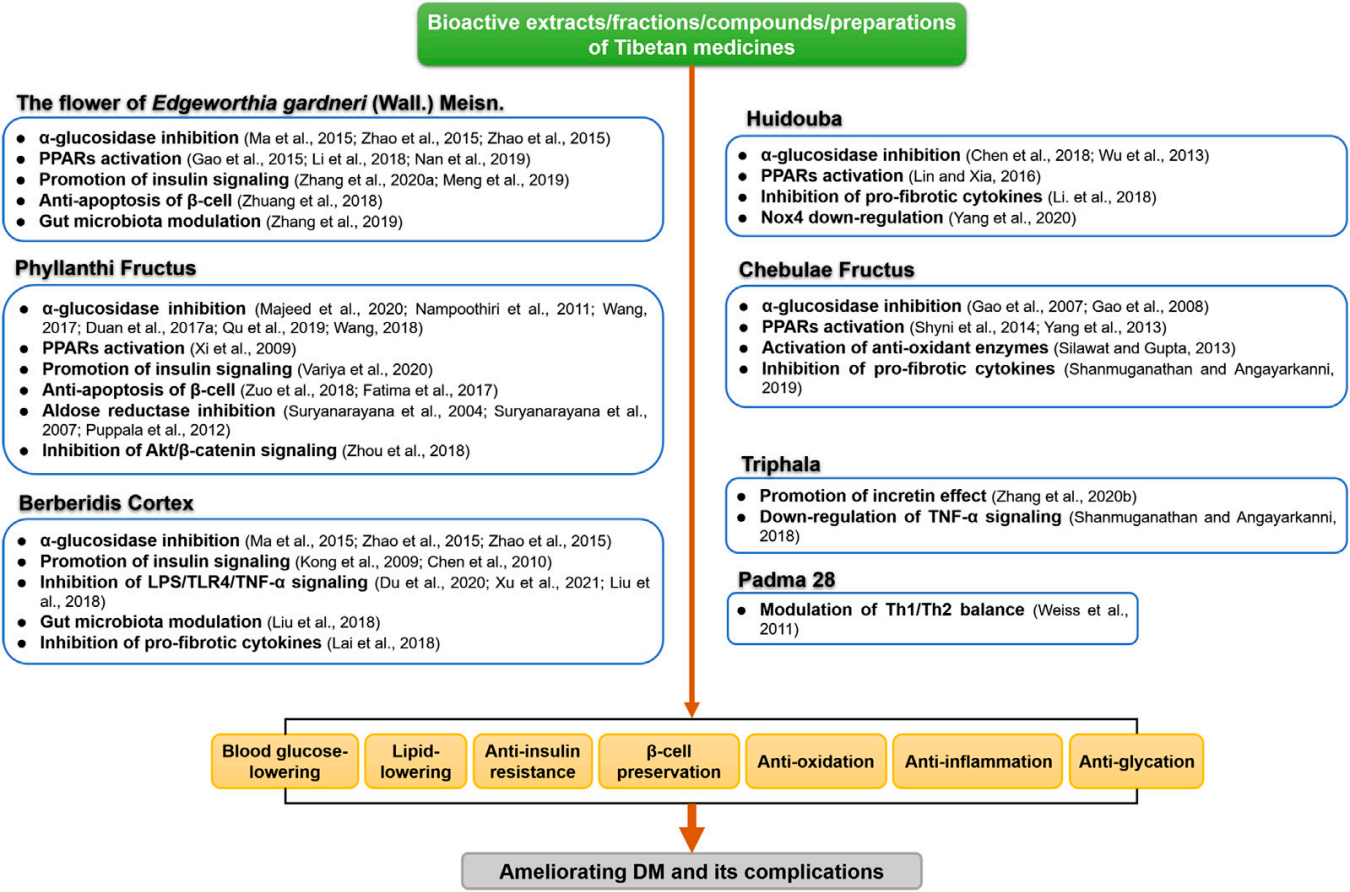
Mechanisms of action of commonly studied anti-DM Tibetan medicines.

**FIGURE 3:**
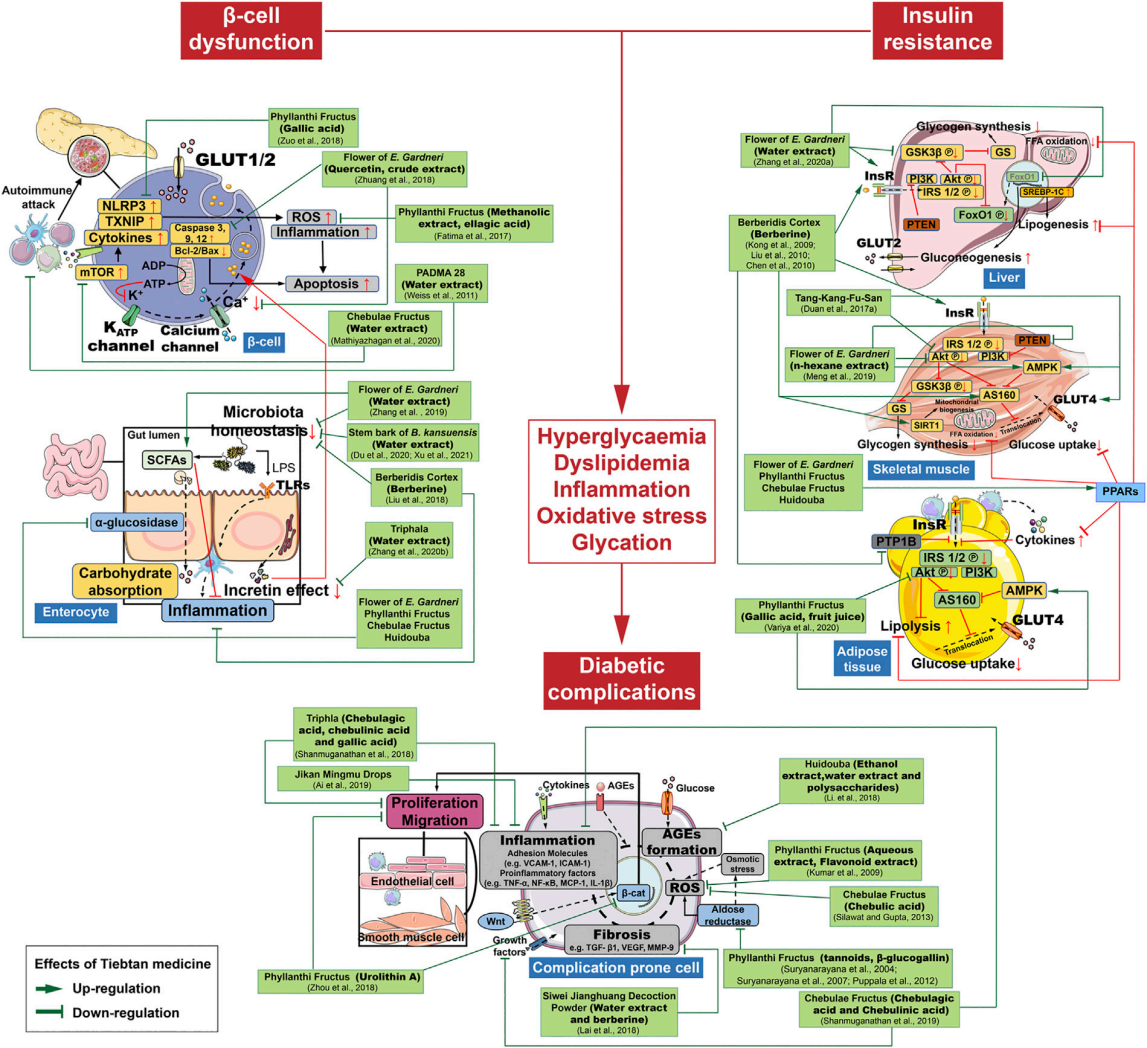
Schematic diagram showing the possible molecular targets of specific bioactive extracts and components from Tibetan medicines.

Our current review summarizes the therapeutic effects of the most commonly studied anti-diabetic TMM in various preclinical and clinical settings. A number of studies centered on mechanisms investigation have indicated that multicomponent TMM acts on multiple molecular targets and signaling pathways to treat DM and its complications. We summarized the possible anti-DM mechanisms and targets of the most commonly used TMM in Figures 2, 3. Supplementary Tables S1, S2 provide a recapitulate of researches of the most studied TTM on DM and its complications.

However, the current researches of TMM on DM and its complications have some limitations. Firstly, most recent studies mainly focus on the extracts/preparations of TMM, and more potent bioactive compounds and fractions isolated from TMM should be studied. Secondly, bioavailability and pharmacokinetics of these active extracts/preparations/compounds should be conducted to provide more information for clinical trials. Thirdly, regarding the diversity of TMM components and the complex pathophysiology of DM, some new technologies, such as genomics, proteomics and metabolomics should be used to systematically evaluate the underlying mechanism of its anti-DM effects. Finally, as DM is a chronic disease with a group of metabolic disorders, long-term use of TMM to maintain stable blood glucose and prevent DM-related is necessary. However, lack of toxicity studies could not ensure the safety and tolerance of TMM. Thus, preclinical studies of acute and chronic toxicity tests and follow-up studies should be designed for TTM researches.

In conclusion, TMM has great potential to be developed into new anti-diabetic agents. Further studies on TTM will definitely extend their clinical applications. With a great respect to previously review about TMM for the treatment of DM (Gao et al., 2021), our review presents an updated pharmacological view of TMM for further exploration and application in DM and its complications treatment, which providing broadened understanding of the underlying mechanisms of TMM to find innovative strategies for DM management.
