# Supplementary material for: Tibetan Medicine for Diabetes Mellitus: Overview of Pharmacological Perspectives
Source: Front Pharmacol. 2021 Oct 21;12:748500. doi: 10.3389/fphar.2021.748500 (PMC8566911; doi:10.3389/fphar.2021.748500)
Supplement: Supplementary file 1 [file DataSheet1.docx]

**Supplementary Materials**

This file includes:

Table S1. Anti-diabetic effects of some commonly used Tibetan medicines;

Table S2. Amelioration of diabetic complications by some commonly used Tibetan medicines;

List of 195 collected DM-TMM-related research articles (from Jan 1st 2000 to June 2021).

**Table S1 Anti-diabetic effects of some commonly used Tibetan medicines.**

| TMM | Origins and used part | Analysts | Models | Intervention | Outcomes | Mechanisms of action | Ref. |
| --- | --- | --- | --- | --- | --- | --- | --- |
| Lv-luo-hua | Flower of *Edgeworthia gardneri* (Wall.) Meisn. | Ethyl acetate extract | (*In vitro*) α-glucosidase/α-amylase | 25 µg/mL | Inhibitory effect (%) =70.87% | Digestive enzymes inhibition | ([Geng et al., 2013](#_ENREF_12)) |
|  |  | Tiliroside |  | - | IC_50_=202 µg/mL |  | ([Ma et al., 2015](#_ENREF_22)) |
|  |  | Edgeworin |  | - | IC_50_=18.7 µg/mL |  | ([Zhao et al., 2015](#_ENREF_51)) |
|  |  | Daphnoretin |  | - | IC_50_=86 µg/mL (α-glucosidase)  IC_50_=90 µg/mL (α-amylase) |  | ([Zhao et al., 2015](#_ENREF_51)) |
|  |  | Ethyl acetate extract; n-hexane extract; n-butanol extract; umbelliferone; pentadecanoic acid | (*In vitro*) HeLa cells transiently co-transfected with reported gene | 3.125-800 µg/mL, for 24 hours | Activated both PPARγ and PPARβ | Acted as dual agonists for PPARγ/β | ([Gao et al., 2015](#_ENREF_9)) |
|  |  | Tiliroside; daphnoretin; pentadecanoic acid | (*In vitro*) high insulin (1μM)-induced HepG2 cell model of insulin resistance | 80 μM for 24 hours | PPARγ, PPARβ and PPARα protein expression ↑ | Acted as agonists for PPARα/γ/β | ([Li et al., 2018](#_ENREF_18)) |
|  |  | Daphnoretin; gardenrd A; daphnoretin-5-O-β-D-glucopyranosyl-(1→2)-O-β-D-glucopyra­noside | (*In vitro*) high insulin (0.1μM)-induced 3T3-L1 cell model of insulin resistance |  |  |  | ([Nan et al., 2019](#_ENREF_27)) |
|  |  | Water extract | (*In vitro*) PA-exposed HepG2 cells | 200-300 μg/mL for 24 hours | Glucose uptake ↑  Glycogen synthesis ↑  Gluconeogenesis ↓ | Regulated IRS1/GSK3β/FoxO1 signaling pathway | ([Zhang et al., 2020a](#_ENREF_48)) |
|  |  | Water extract | (*In vivo*) HFD and STZ-induced T2DM C57BL/6J mice | p.o. 1-3 g/kg BW per day for 4 weeks | Glucose tolerance ↑  HOMA-IR index ↓  Improved lipid metabolism | Modulated gut bacterial phylotypes | ([Zhang et al., 2019](#_ENREF_50)) |
|  |  | Fraction 1 from n-hexane extract | (*In vitro*) PA-exposed C2C12 cells;  (*in vivo*) *db/db* mice; HFD and STZ-induced T2DM C57BL/6J mice | (*In vitro*) 200-300 μg/mL for 24 hours;  (*In vivo*) p.o. 40-160 mg/kg BW per day for 4 weeks | Glucose uptake ↑  Glucose tolerance ↑  HOMA-β index ↑  HOMA-IR index ↓  HbA1c ↓ | Activated PI3K/Akt/AMPK signaling pathway | ([Meng et al., 2019](#_ENREF_25)) |
|  |  | Quercetin isolated from ethyl acetate fraction; crude extract | (*In vitro*) PA-exposed MIN-6 cells;  (*In vivo*) *db/db* mice | (*In vitro*) 10 μM for 24 hours;  (*in vivo*) p.o. 0.5 g/kg BW quercetin per day for 4 weeks | Insulin secretion ↑  Cell apoptosis ↓  Damage in pancreatic islets and livers ↓ | Enhanced insulin secretion via calcium and ERK1/2 signaling pathway; anti-apoptosis of β-cell by inhibiting caspase-3, -9, -12; increasing the ratio of Bcl-2/BAX and reversing impaired mitochondrial membrane potential | ([Zhuang et al., 2018](#_ENREF_55)) |
| Skyu-ru-ra | Fruits of *Phyllanthus emblica* L. | Fruit extract containing 𝛽-glucogallin along with hydrolysable tannins | (*In vitro*) porcine pancreatic α-amylase, human salivary α-amylase and yeast α-glucosidase | - | IC_50_=135.7 µg/mL (pancreatic α-amylase)  IC_50_=106.7 µg/mL (salivary α-amylase)  IC_50_=562.9 µg/mL (α-glucosidase) | Digestive enzymes inhibition | ([Majeed et al., 2020](#_ENREF_23)) |
|  |  | Methanol extract | (*In vitro*) α-amylase, α-glucosidase and albumin-glucose complex |  | IC_50_=94.3 µg/mL (α-amylase)  IC_50_=1.0 µg/mL (α-glucosidase)  IC_50_=182.9 µg/mL (glycation) | Digestive enzymes inhibition; anti-glycation | ([Nampoothiri et al., 2011](#_ENREF_26)) |
|  |  | Polyphenol extract | (*In vitro*) α-glucosidase |  | IC_50_=710 µg/mL | Digestive enzymes inhibition | ([Wang, 2017](#_ENREF_39)) |
|  |  | Phenolic constituents |  | - | IC_50_=286-1680μM |  | ([Duan et al., 2017a](#_ENREF_6)) |
|  |  | Corilagin |  | - | IC_50_=9.7 µg/mL |  | ([Qu et al., 2019](#_ENREF_29)) |
|  |  | Polysaccha­rides | (*In vitro*) α-amylase and α-glucosidase | - | IC_50_=1670 µg/mL (α-amylase)  IC_50_=890 µg/mL (α-glucosidase) |  | ([Wang, 2018](#_ENREF_40)) |
|  |  | Water extract with alcohol precipitation | (*In vivo*) HFD-induced obese Wistar rats | p.o. 3.5 g/kg BW per day for 6 weeks | Body weight, FBG ↓  HOMA-IR index ↓  Improved lipid metabolism  PPARγ mRNA, adiponectin ↑ | PPARγ activation | ([Xi et al., 2009](#_ENREF_43)) |
|  |  | Gallic acid; fruit juice | (*In vitro*) 3T3-L1 cells;  (*In vivo*) *db/db* mice and fructose-induced metabolic maladies SD rats | (*In vitro*) 10-20 mM;  (*in vivo*) p.o. gallic acid at a dose of 100 mg/kg/day, or fruit juice at a dose of 2 ml/kg/day for 42 days (*db/db* mice) or for 4 weeks (fructose-induced metabolic maladies SD rats) | 3T3-L1 differentiation ↑  Glucose uptake ↑  HOMA-IR index ↓  Body weight, glucose and lipid homeostasis ↑  Hemodynamic pressure response ↓ | Activated PPARγ and Akt/AMPK signaling to improved glucose transporters and insulin sensitivity | ([Variya et al., 2020](#_ENREF_38)) |
|  |  | Gallic acid | (*In vitro*) high-glucose (25mM)-treated INS-1 cells;  (*In vivo*) STZ-induced diabetic Wistar rats | (*In vitro*) 2.5-10 μM for 48 hours;  (*in vivo*) p.o. 50 mg/kg BW/day for 4 weeks | Cell apoptosis ↓  Islet morphology and structural integrity ↑  TXNIP, NLRP3 ↓ | Anti-apoptosis of β-cell via downregulation of TXNIP, NLRP3 expression | ([Zuo et al., 2018](#_ENREF_56)) |
|  |  | Methanolic extract; ellagic acid | (*In vitro*) isolated mouse islets;  (*in vivo*) neonatal STZ-induced non-obese type 2 diabetic rats | (*In vitro*) 50-200 μM for 60min;  (*in vivo*) ellagic acid at 25-100 mg/kg/day, or the methanolic extract at 250-500 mg/kg/day for 28 days | Insulin secretion ↑  Glucose tolerance ↑  Antioxidant capacity ↑  Β-cell mass ↑ | Preservation of β-cell mass and function by ameliorating oxidative stress | ([Fatima et al., 2017](#_ENREF_8)) |
| A-ru-ra/ Haritaki | Fruits of *Terminalia chebula* Retz. | Ethyl acetate (TC-2),  methanol (TC-3) and 70%methanol-30%water extract (TC-4) extracts | (*In vitro*) α-glucosidase | - | IC_50_=0.19 µg/mL (TC-2)  IC_50_=0.28 µg/mL (TC-3)  IC_50_=1.81 µg/mL (TC-4) | Digestive enzymes inhibition | ([Sasidharan et al., 2012](#_ENREF_30)) |
|  |  | 70% methanol extract from the young fruits | (*In vitro*) rat small intestine and Caco-2 cells; (*in vivo*) sucrose/maltose-challenged rats | (*In vitro*) 0.5 μg/mL  (*In vivo*) p.o. 500mg/kg BW 5min before challenge | IC50=100 µg/mL (in small intestinal model)  Inhibitory effect (%) =80% (in Caco-2 cells) | Selectively inhibited maltase | ([Jin et al., 2010](#_ENREF_14)) |
|  |  | chebulagic acid (2), chebulinic acid (3) and 1,2,3,4,6-Penta-O-galloyl-β-D-glucose (4) | (*In vitro*) α-glucosidase for the hydrolysis of maltose | - | IC_50_=93 µg/mL (2)  IC_50_=34 µg/mL (3)  IC_50_=132 µg/mL (4) |  | ([Gao et al., 2007](#_ENREF_11)) |
|  |  | Chebulagic acid | (*In vitro*) maltase-glucoamylase (M-G) complex | - | IC_50_=10.3 µg/mL |  | ([Gao et al., 2008](#_ENREF_10)) |
|  |  | Chebulagic acid | (*In vitro*) 3T3-L1 cells | 10-100 μM | Glucose uptake ↑  Lipid accumulation ↓  PPARγ, GLUT4, adiponectin ↑ | Acted as a partial PPARγ agonist | ([Shyni et al., 2014](#_ENREF_33)) |
|  |  | 2,3,6-tri-*O*-galloyl-β-D-glucose, 1,2,3,6-tetra-*O*-galloyl-β-D-glucose and 1,2,3,4,6-penta-*O*-galloyl-β-D-glucose | (*In vitro*) HepG2 cells transfected with reporter gene and 3T3-L1 cells | 25-100 μM for HepG2 cells; 3-30 μM for 3T3-L1 cells | PPARα/γ activity ↑  Glucose uptake ↑  Lipid accumulation ↓ | Acted as partial PPARγ agonists without inducing adipogenesis | ([Yang et al., 2013](#_ENREF_46)) |
|  |  | Decoction lyophilized powder | (*In vitro*) fructose and STZ-induced diabetic rats | 400 mg/kg BW/day for 28 days | Blood glucose ↓  HOMA-β index ↑  HOMA-IR index ↓  Liver function ↑  Pancreatic damage and mTOR ↓ | mTOR inhibition | ([Mathiyazhagan and Kodiveri Muthukaliannan, 2020](#_ENREF_24)) |
| Huidou-ba | The strip-shaped or bag-shaped cobwebs of spiders of the genus Atypus which lived on tea plants in Mount Emei | Polysaccharide fractions | (*In vitro*) α-glucosidase | - | CHBDP: IC50=19 µg/mL  HBDP-1 (127.33 kDa): IC50=39 µg/mL  HBDP-2 (9.703 kDa): IC50=13 µg/mL  HBDP-3A (86749 kDa): IC50=7.5 µg/mL | Digestive enzymes inhibition | ([Chen et al., 2018a](#_ENREF_3); [Chen et al., 2018b](#_ENREF_4)) |
|  |  | Protein fraction | (*In vitro*) α-glucosidase | 10 mg/mL | Inhibitory effect (%) =46. 84% | Digestive enzymes inhibition | ([Wu et al., 2013](#_ENREF_42)) |
|  |  | Ethyl acetate extract and n-hexane extract | (*in vitro*) HeLa cells transfected with reporter gene | 50-200μM | Activated both PPARγ and PPARβ | Acted as dual agonists for PPARγ/β | ([Lin and Xia, 2016](#_ENREF_19)) |
| Cortex Berberi-dis | The dried root or stem bark of *Berberis kansuensis* Schneid. and its relative plants (*B. vernae* Schneid. and *B. dictyophylla* Franch.) | Water extract of the dried stem bark of *B. kansuensis* | (*In vivo*) HFD and STZ-induced T2DM rats | 0.84 g/kg (crude herb equivalent) BW/day for 30 days | FBG, GSP, insulin, HOMA-IR index, LPS, TNF-α and IL-6 ↓  Insulin sensitive index ↑ | Anti-inflammation; alleviated insulin resistance; restored disturbed metabolic pathways and regulated gut microbiota | ([Du et al., 2020](#_ENREF_5); [Xu et al., 2021](#_ENREF_44)) |
|  |  | Berberine | (*In vitro*) HepG2, Bel-7402 human liver cell lines and L6 rat skeletal muscle cells;  (*in vivo*) HFD and STZ-induced T2DM Waster rats and KK-Ay T2DM mice | (*In vitro*) 7.5 μg/mL;  (*in vivo*) p.o. 75 or 150 mg/kg BW/day for 15 days (rats); p.o. 100 or 200 mg/kg/day BW for 3 weeks (mice) | Glucose consumption ↑  Insulin receptor mRNA ↑  Fasting blood glucose and fasting serum insulin ↓  PKC activity ↑ | Increased IR gene expression through a protein kinase C (PKC)–dependent activation of its promoter both in liver and skeletal muscle | ([Kong et al., 2009](#_ENREF_15)) |
|  |  |  | (*In vitro*) 3T3-L1 adipocytes and L6 myocytes  (*In vivo*) *db/db* mice and HFD-induced obese mice | (*In vitro*) 1.25-20 μM  (*In vivo*) p.o. 100 mg/kg BW per day at 2:00 p.m. for 2 weeks | Glucose uptake ↑  Glucose tolerance ↑  Phosphorylation of IRS1 (Ser307), Akt (Ser473) and Akt (Ser308) ↑  PTP1B phosphatase activity ↓ | Insulin-mimicry effects via inhibition of PTP1B activity | ([Chen et al., 2010](#_ENREF_2)) |
|  |  |  | (*In vivo*) HFD-fed obese rats | p.o. 200 mg/kg BW/day for 8 weeks | Improved metabolic and biochemical parameters  TLR4, TNF-α ↓  IR, IRS-1 mRNA ↑  Microbiota composition change ↓  Hepatic steatosis ↓ | Modulated gut bacterial phylotypes and inhibited LPS/TLR4/TNF-α signaling | ([Liu et al., 2018](#_ENREF_20)) |
|  |  |  | (*In vitro*) high glucose or FFA-stimulated C2C12 cells;  (*in vivo*) HFD-fed SD rats | (*In vitro*) 5μM  (*In vivo*) p.o. 100 mg/kg BW per day for 4 weeks | Glucose tolerance ↑  Leptin/Adiponectin ratio ↓  Mitochondrial function ↑ | Activated SIRT1-dependent mitochondrial biogenesis | ([Gomes et al., 2012](#_ENREF_13)) |
|  |  |  | (*in vitro*) high glucose (25mM) and high insulin (100 nM)-exposed L6 cells | 5 μg/mL | Glucose uptake ↑  GLUT4 translocation ↑  AS160 phosphorylation ↑  Actin remodeling ↑ | Activated AMPK/AS160/GLUT4 signaling pathway | ([Liu et al., 2010](#_ENREF_21)) |
| Tripha-la | Equal portion of the fruits from *P. emblica*, *T. chebula* and *Terminalia bellirica* (Gaertn.) Roxb. | Water extract | (*In vivo*) STZ-induced diabetic rats | p.o. 0.43, 0.86 or 1.72g/kg BW/day for 6 weeks | Blood glucose ↓  Insulin secretion ↑  Islet atrophy ↓  Islet β-cell ↑  Incretins ↑ | Activated PKA-dependent incretin/cAMP signaling pathway | ([Zhang et al., 2020b](#_ENREF_49)) |
| Padma 28 | 22 herbal drugs and calcium sulfate | Water extract lyophilized powder | (*In vivo*) female non-obese diabetic (NOD) mice | i.p. 0.3 mL (3 mg dry weight of Padma 28) for 13 weeks. | Development of overt diabetes ↓  Glucose tolerance ↑  Insulin secretion ↑  Th2 cytokines ↑  CD8 T cells ↓ | Immunomodulatory effects associated with a shift from Th1 to Th2 immune response | ([Weiss et al., 2011](#_ENREF_41)) |
| Tang-Kang-Fu-San | 11 medicinal herbs including *Berberis kansuensis* Schneid., *Curcuma longa* L., *Phyllanthus emblica* L. etc. | Prepared formula | (*In vivo*) *db/db* mice | p.o. 1-2 g/kg BW/day for 4 weeks | Glucose tolerance ↑  Insulin tolerance ↑  HOMA-IR ↓  p-Akt, p-AMPK and GLUT4 ↑ | Improved insulin resistance by activating PI3K/Akt and AMPK/GLUT signaling | ([Duan et al., 2017a](#_ENREF_6); [Duan et al., 2017b](#_ENREF_7)) |

**Table S2 Amelioration of diabetic complications by some commonly used Tibetan medicines.**

| Complications | TMM | Analysts | Models | Intervention | Outcomes | Mechanisms of action | Ref. |
| --- | --- | --- | --- | --- | --- | --- | --- |
| Diabetic nephropathy | Huidouba | Ethanol extract, water extract and polysaccharides | (*In vivo*) STZ and HFD-induced T2DM nephropathic mice | p.o. 100-200 mg/kg BW per day for 8 weeks | Blood glucose ↓  Renal tissue damage ↓  Urine protein ↓  SCr ↓ BUN ↓ GSP ↓ AGEs ↓  FN ↓ ICAM-1 ↓ TGF-β1 ↓ | Inhibited formation of AGEs and anti-fibrosis | ([Zhou et al., 2018b](#_ENREF_54)) |
|  |  | Aqueous extract | (*In vivo*) STZ and HFD-induced T2DM nephropathic mice | p.o. 100 mg/kg BW per day for 3 weeks | Glucose tolerance ↑  Renal tissue damage ↓  podocyte loss ↓  Urine protein ↓  SCr ↓ BUN ↓ MDA ↓ | Anti-oxidation via downregulating Nox4 expression | ([Yang et al., 2020](#_ENREF_45)) |
|  | Chebulae Fructus | Chebulic acid | (*In vitro*) STZ-induced diabetic rats with renal ischemia/reperfusion unilaterally | p.o. 25-50 mg/kg/day for 28 days | Biochemical parameters ↑  Glomerular damage ↓  Mesangial matrix increment ↓  Basal membrane thickening ↓  Antioxidative capacity ↑ | Ameliorated oxidative stress | ([Silawat and Gupta, 2013](#_ENREF_34)) |
|  | Siwei Jianghuang Decoction Powder: Curcumae Longae Rhizoma, Berberidis Cortex, Phyllanthi Fructus, Tribuli Fructus (1:2:1:2) | Water extract and berberine | (*In vivo*) *db/db* mice | p.o. 0.978-3.914 g/kg BW per day for 8 weeks (BBR: 0.157 g/kg BW) | Blood glucose ↓  Renal fibrosis ↓  Urine protein ↓ SCr ↓ BUN ↓  Urine microalbumin ↓  Serum uric acid ↓  Urinary albumin excretion ↓ | Down-regulated HIF-1α, VEGF, TGF-β1 overexpression | ([Lai et al., 2018](#_ENREF_17)) |
| Diabetic retinopathy | Phyllanthi Fructus | Enriched tannoids fraction | (*In vitro*) rat lens and recombinant human aldose reductase; *Ex vivo*, sugar-exposed cultured rat lens organ; | Ex vivo: 50 µg/mL | IC_50_=6 µg/mL (rat lens AR)  IC_50_=10 µg/mL (human AR)  Osmotic changes ↓ | Inhibition of aldose reductase | ([Suryanarayana et al., 2004](#_ENREF_35)) |
|  |  | Enriched tannoids fraction | (*In vivo*) STZ-induced diabetic rats | Administrated with AIN-93 diet containing 0.2% tannoids mixture for 8 weeks | Delayed cataract progression  Aldose reductase activity ↓  Sorbitol ↓  Antioxidant enzymes activity ↑  Aggregation and insolubilization of lens proteins ↓ | Inhibition of aldose reductase and anti-oxidation | ([Suryanarayana et al., 2007](#_ENREF_36)) |
|  |  | β-glucogallin | (*In vitro*) human aldose reductase; *Ex vivo,* cultured lens from transgenic mice overexpressing human AR in the lens | (*Ex vivo*) 30 µM | IC_50_=17 µM  Hyperglycemia-induced sorbitol accumulation ↓ | Inhibition of aldose reductase | ([Puppala et al., 2012](#_ENREF_28)) |
|  | Cortex Berberis dictyophlla | Aqueous extract and Berberine | (*In vivo*) *db/db* mice | p.o. 0.38-1.5 g/kg BW per day for 60 days (BBR: 0.135 g/kg BW) | Blood glucose ↓  Retinal damage ↓  Endotheliocyte/Pericyte ratio ↓ | Down-regulated HIF-1α, PKC-β, VEGF | ([Ye et al., 2016](#_ENREF_47); [Zhou et al., 2016](#_ENREF_52)) |
|  | Chebulae Fructus | Chebulagic acid and chebulinic acid | (*In vivo*) TGFβ1-exposed RF/6A cells | 10 μM for 48hours | Pro-fibrotic factor expression ↓  Cell proliferation and migration ↓  ERK phosphorylation ↓  Binding with TGFβ receptor in silico docking studies | Inhibited fibrotic transformation via inhibition of ERK phosphorylation and TGFβ signaling | ([Shanmuganathan and Angayarkanni, 2019](#_ENREF_32)) |
|  | Jikan Mingmu Drops: *B. dictyophylla* Cortex, Chebulae Fructus, Carthami Flos, Borneolum Syntheticum, tauroursodeoxycholic acid, and muscone (100:200:200:20:0.15:0.1) | Prepared Jikan Mingmu Drops with sterilization | (*In vivo*) benzalkonium chloride (BAC)-induced DES model of *db/db* mice | Topical administration of 5μL JMD (0.25-1.0g/mL) three times daily (9 a.m., 3 p.m., and 9 p.m.) for 7 days | Tear volume ↑  Corneal damage ↓  Goblet cells ↑  Proinflammatory cytokines ↓ | Anti-inflammation | ([Ai et al., 2019](#_ENREF_1)) |
|  | Triphala [Equal portion of the fruits from *P. emblica*, *T. chebula* and *Terminalia bellirica* (Gaertn.) Roxb.] | Chebulagic acid, chebulinic acid and gallic acid | (*In vitro*) TNFα-exposed RF/6A cells; | 1-100 μM | Angiogenesis ↓  Pro-inflammatory cytokines ↓  Pro-angiogenic MMP-9 ↓  Cell proliferation and migration ↓  Phosphorylation of p38, ERK and NF-κB ↓  Binding with TNFα receptor in silico docking studies | Anti-angiogenesis and anti-inflammation via inhibition of p38, ERK and NF-κB phosphorylation and TNF-α signaling | ([Shanmuganathan and Angayarkanni, 2018](#_ENREF_31)) |
| Diabetic neuropathy | Phyllanthi Fructus | Aqueous extract | (*In vivo*) STZ-induced diabetic rats | p.o. 250, 500 or 1000 mg/kg/day for 4 weeks | Blood glucose ↓  Nociceptive threshold ↑  Lipid peroxidation ↓  Nitrative and oxidative stress in sciatic nerve↓  Pro-inflammatory cytokines ↓ | Relieved neuropathic pain through  modulation of oxidative-nitrative stress | ([Tiwari et al., 2011](#_ENREF_37)) |
|  |  | Flavonoid rich fruit extract (ethyl acetate: methanol fraction) | (*In vivo*) HFD and STZ-induced diabetic rats | p.o. 10 mg/kg BW/day for 8 weeks | Glucose tolerance ↑  Nociceptive threshold ↑  Oxidative stress in sciatic nerve ↓  Axonal degeneration ↓ | Anti-oxidation | ([Kumar et al., 2009](#_ENREF_16)) |
| Macrovascu­lar complication | Phyllanthi Fructus | Fruit juice and its metabolite urolithin A | (*In vitro*) glucose (30 mM) or LiCl (20 mM)-stimulated A7r5 vascular smooth muscle cells;  (*in vivo*) STZ-induced diabetic rats | (*In vitro*) 5-40 μM for 48 hours;  (*in vivo*) p.o. 25-75 g/kg of original fruit samples per day for 5 weeks | Akt (Thr308) phosphorylation ↓  β-catenin ↓  c-Myc and cyclin D1 ↓  Vascular smooth muscle cell proliferation ↓ | Down-regulated Akt/β-catenin signaling | ([Zhou et al., 2018a](#_ENREF_53)) |

**References:**

Ai, X.P., Hou, Y., Wang, X.B., Wang, X.Y., Liang, Y.S., Zhu, Z.W., et al. (2019). Amelioration of dry eye syndrome in db/db mice with diabetes mellitus by treatment with Tibetan Medicine Formula Jikan Mingmu Drops. *J Ethnopharmacol* 241**,** 111992.

Chen, C.H., Zhang, Y.B., and Huang, C. (2010). Berberine inhibits PTP1B activity and mimics insulin action. *Biochem Biophys Res Commun* 397(3)**,** 543-547. doi: 10.1016/j.bbrc.2010.05.153.

Chen, J., Li, L., Zhou, X., Li, B., Zhang, X., and Hui, R. (2018a). Structural characterization and α-glucosidase inhibitory activity of polysaccharides extracted from Chinese traditional medicine Huidouba. *Int J Biol Macromol* 117**,** 815-819. doi: 10.1016/j.ijbiomac.2018.05.192.

Chen, J.C., Li, L., Zhou, X., Sun, P.Y., Li, B., and Zhang, X. (2018b). Preliminary characterization and antioxidant and hypoglycemic activities in vivo of polysaccharides from Huidouba. *Food Func* 9(12)**,** 6337-6348. doi: 10.1039/c8fo01117f.

Du, H., Li, Q., Yi, H., Xu, T., Xu, X.M., Kuang, T.T., et al. (2020). Anti-Diabetic Effects of Berberis kansuensis Extract on Type 2 Diabetic Rats Revealed by (1) H-NMR-Based Metabolomics and Biochemistry Analysis. *Chem Biodivers* 17(10)**,** e2000413. doi: 10.1002/cbdv.202000413.

Duan, B.L., Zhao, Z.Q., Liao, W.F., Xiong, H., Liu, S.S., Yin, L., et al. (2017a). Antidiabetic Effect of Tibetan Medicine Tang-Kang-Fu-San in db/db Mice via Activation of PI3K/Akt and AMPK Pathways. *Front Pharmacol* 8. doi: 10.3389/fphar.2017.00535.

Duan, B.L., Zhao, Z.Q., Lin, L., Jin, J., Zhang, L.J., Xiong, H., et al. (2017b). Antidiabetic Effect of Tibetan Medicine Tang-Kang-Fu-San on High-Fat Diet and Streptozotocin-Induced Type 2 Diabetic Rats. *Evid Based Complement Alternat Med* 2017(7302965)**,** 1-9. doi: 10.1155/2017/7302965.

Fatima, N., Hafizur, R.M., Hameed, A., Ahmed, S., Nisar, M., and Kabir, N. (2017). Ellagic acid in *Emblica officinalis* exerts anti-diabetic activity through the action on β-cells of pancreas. *Eur J Nutr* 56(2)**,** 591-601. doi: 10.1007/s00394-015-1103-y.

Gao, D., Zhang, Y.L., Xu, P., Lin, Y.X., Yang, F.Q., Liu, J.H., et al. (2015). In vitro evaluation of dual agonists for PPARγ/β from the flower of Edgeworthia gardneri (wall.) Meisn. *J Ethnopharmacol* 162**,** 14-19. doi: 10.1016/j.jep.2014.12.034.

Gao, H., Huang, Y.N., Gao, B., and Kawabata, J. (2008). Chebulagic acid is a potent α-glucosidase inhibitor. *Biosci Biotechnol Biochem* 72(2)**,** 601-603. doi: 10.1271/bbb.70591.

Gao, H., Huang, Y.N., Xu, P.Y., and Kawabata, J. (2007). Inhibitory effect on α-glucosidase by the fruits of *Terminalia chebula* Retz. *Food Chem* 105(2)**,** 628-634. doi: 10.1016/j.foodchem.2007.04.023.

Geng, Y., Yang, H.M., Xu, H.Y., and Shi, J.-S. (2013). α-Glucosidase inhibitory activity of the alabastrum of Edgeworthia gardneri (Wall.) Meissn. *J Food Sci Biotechnol* 32**,** 967-971.

Gomes, A., Duarte, F.V., Nunes, P.M., Hubbard, B.P., Teodoro, J.S., Varela, A.T., et al. (2012). Berberine protects against high fat diet-induced dysfunction in muscle mitochondria by inducing SIRT1-dependent mitochondrial biogenesis. *Biochim Biophys Acta* 1822(2)**,** 185-195. doi: 10.1016/j.bbadis.2011.10.008.

Jin, Z., Zeng, W.C., Luo, J.W., Ye, H.X., Huang, Y.N., and Gao, H. (2010). In vitro and in vivo inhibitory effect of methanol extract from *Terminalia chebula* Retz. Fruits on α-Glucosidase. *Food Sci* 31(07)**,** 284-287. doi: 10.7506/spkx1002-6300-201007063.

Kong, W.J., Zhang, H., Song, D.Q., Xue, R., Zhao, W., Wei, J., et al. (2009). Berberine reduces insulin resistance through protein kinase C–dependent up-regulation of insulin receptor expression. *Metabolism* 58(1)**,** 109-119.

Kumar, N.P., Annamalai, A.R., and Thakur, R.S. (2009). Antinociceptive property of *Emblica officinalis* Gaertn (Amla) in high fat diet-fed/low dose streptozotocin induced diabetic neuropathy in rats. *Indian J Exp Biol* 47(9)**,** 737-742.

Lai, X., Tong, D., Ai, X., Wu, J., Luo, Y., Zuo, F., et al. (2018). Amelioration of diabetic nephropathy in db/db mice treated with tibetan medicine formula Siwei Jianghuang Decoction Powder extract. *Sci Rep* 8(1)**,** 16707. doi: 10.1038/s41598-018-35148-2.

Li, M., Nan, C.Y., Zhu, J.X., and Zhong, G.C. (2018). Screening of anti-T2DM PPARs agonist from Lvluohua. *Chin Tradit Pat Med* 40(10)**,** 2285-2288. doi: 10.3969 /j.issn.1001-1528.2018.10.034.

Lin, Y.X., and Xia, Z.N. (2016). Screening Active Fractions of Peroxisome Proliferator Activated Receptor γ in Natural Medicines with Potential Antidiabetic Activity. *Nat Prod Res Dev* 28(04)**,** 505-513.

Liu, D., Zhang, Y., Liu, Y., Hou, L., Li, S., Tian, H., et al. (2018). Berberine Modulates Gut Microbiota and Reduces Insulin Resistance via the TLR4 Signaling Pathway. *Exp Clin Endocrinol Diabetes* 126(8)**,** 513-520. doi: 10.1055/s-0043-125066.

Liu, L.Z., Cheung, S., Lan, L.L., Ho, S.K.S., Xu, H.X., Chan, J.C.N., et al. (2010). Berberine modulates insulin signaling transduction in insulin-resistant cells. *Mol Cell Endocrinol* 317(1)**,** 148-153. doi: 10.1016/j.mce.2009.12.027.

Ma, Y.Y., Zhao, D.G., Zhou, A.Y., Zhang, Y., Du, Z., and Zhang, K. (2015). α-Glucosidase Inhibition and Antihyperglycemic Activity of Phenolics from the Flowers of Edgeworthia gardneri. *J Agric Food Chem* 63(37)**,** 8162-8169. doi: 10.1021/acs.jafc.5b03081.

Majeed, M., Majeed, S., Mundkur, L., Nagabhushanam, K., Arumugam, S., Beede, K., et al. (2020). Standardized *Emblica officinalis* fruit extract inhibited the activities of α-amylase, α-glucosidase, and dipeptidyl peptidase-4 and displayed antioxidant potential. *J Sci Food Agric* 100(2)**,** 509-516. doi: 10.1002/jsfa.10020.

Mathiyazhagan, J., and Kodiveri Muthukaliannan, G. (2020). The role of mTOR and oral intervention of combined Zingiber officinale-Terminalia chebula extract in type 2 diabetes rat models. *J Food Biochem***,** e13250. doi: 10.1111/jfbc.13250.

Meng, Z.M., Geng, Y., Li, H., Xu, H.Y., Zhao, H., Liu, M., et al. (2019). Ameliorative Effects of Fraction 1 from the Flower of *Edgeworthia gardneri* (Wall.) Meisn on Insulin Resistance of C2C12 Cells. *J Food Sci Biotechnol* 38(11)**,** 55-62. doi: 10.3969/j.issn.1673-1689.2019.11.008.

Nampoothiri, S.V., Prathapan, A., Cherian, O.L., Raghu, K.G., Venugopalan, V.V., and Sundaresan, A. (2011). In vitro antioxidant and inhibitory potential of Terminalia bellerica and *Emblica officinalis* fruits against LDL oxidation and key enzymes linked to type 2 diabetes. *Food Chem Toxicol* 49(1)**,** 125-131. doi: 10.1016/j.fct.2010.10.006.

Nan, C.Y., Zhong, G.Y., Zhu, J.X., JIang, W., and Li, M. (2019). Effects of Components from Edgeworthia gardneri Meissn on PPARs Level in 3T3-L1 Cells. *Tradit Chin Drug Res Pharmaco* 30(03)**,** 282-288. doi: 10.19378/j.issn.1003-9783.2019.03.003.

Puppala, M., Ponder, J., Suryanarayana, P., Reddy, G.B., Petrash, J.M., and LaBarbera, D.V. (2012). The isolation and characterization of β-glucogallin as a novel aldose reductase inhibitor from Emblica officinalis. *PLoS One* 7(4)**,** e31399. doi: 10.1371/journal.pone.0031399.

Qu, Y.Q., Zhao, W.J., Chen, J.G., Yi, Z.P., Li, M.X., and Wan, C.P. (2019). α-glucosidase inhibitory activity of corilagin from *Phyllanthus emblica* L. *Jiangsu Agric Sci* 47(14)**,** 206-209. doi: 10.15889 /j. issn.1002-1302.2019.14. 048.

Sasidharan, I., Sundaresan, A., Nisha, V.M., Kirishna, M.S., Raghu, K.G., and Jayamurthy, P. (2012). Inhibitory effect of Terminalia chebula Retz. fruit extracts on digestive enzyme related to diabetes and oxidative stress. *J Enzyme Inhib Med Chem* 27(4)**,** 578-586. doi: 10.3109/14756366.2011.603130.

Shanmuganathan, S., and Angayarkanni, N. (2018). Chebulagic acid Chebulinic acid and Gallic acid, the active principles of Triphala, inhibit TNFα induced pro-angiogenic and pro-inflammatory activities in retinal capillary endothelial cells by inhibiting p38, ERK and NFkB phosphorylation. *Vascul Pharmacol* 108**,** 23-35. doi: 10.1016/j.vph.2018.04.005.

Shanmuganathan, S., and Angayarkanni, N. (2019). Chebulagic acid and Chebulinic acid inhibit TGF-β1 induced fibrotic changes in the chorio-retinal endothelial cells by inhibiting ERK phosphorylation. *Microvasc Res* 121**,** 14-23. doi: 10.1016/j.mvr.2018.09.001.

Shyni, G.L., Kavitha, S., Indu, S., Arya, A.D., Anusree, S.S., Vineetha, V.P., et al. (2014). Chebulagic acid from Terminalia chebula enhances insulin mediated glucose uptake in 3T3-L1 adipocytes via PPARγ signaling pathway. *Biofactors* 40(6)**,** 646-657. doi: 10.1002/biof.1193.

Silawat, N., and Gupta, V.B. (2013). Chebulic acid attenuates ischemia reperfusion induced biochemical alteration in diabetic rats. *Pharm Biol* 51(1)**,** 23-29. doi: 10.3109/13880209.2012.698288.

Suryanarayana, P., Kumar, P.A., Saraswat, M., Petrash, J.M., and Reddy, G.B. (2004). Inhibition of aldose reductase by tannoid principles of *Emblica officinalis*: implications for the prevention of sugar cataract. *Mol Vis* 10**,** 148-154.

Suryanarayana, P., Saraswat, M., Petrash, J.M., and Reddy, G.B. (2007). Emblica officinalis and its enriched tannoids delay streptozotocin-induced diabetic cataract in rats. *Mol Vis* 13**,** 1291-1297.

Tiwari, V., Kuhad, A., and Chopra, K. (2011). *Emblica officinalis* corrects functional, biochemical and molecular deficits in experimental diabetic neuropathy by targeting the oxido-nitrosative stress mediated inflammatory cascade. *Phytother Res* 25(10)**,** 1527-1536. doi: 10.1002/ptr.3440.

Variya, B.C., Bakrania, A.K., and Patel, S.S. (2020). Antidiabetic potential of gallic acid from Emblica officinalis: Improved glucose transporters and insulin sensitivity through PPAR-γ and Akt signaling. *Phytomedicine* 73**,** 152906. doi: 10.1016/j.phymed.2019.152906.

Wang, R. (2017). Inhibition effect on α-Glucosidase and antioxidant activity for polyphenol extracts from *Phyllanthus emblica* L. *Food Res Dev* 38(11)**,** 13-16. doi: 10.3969/j.issn.1005-6521.2018.17.034.

Wang, R. (2018). Study on hypoglycemic and antioxidant activity of polysaccharide from *Phyllanthus emblica* L. in vitro. *Food Res Dev* 39(17)**,** 189-192+224. doi: 10.3969/j.issn.1005-6521.2017.11.004.

Weiss, L., Barak, V., and Raz, I. (2011). Herbal flavonoids inhibit the development of autoimmune diabetes in NOD mice: proposed mechanisms of action in the example of PADMA 28. *Altern Med Stud* 1(1)**,** e1-e1. doi: 10.4081/ams.2011.e1.

Wu, Y., Wang, X.N., Chen, F.F., Chen, Y.N., and Tian, S.Q. (2013). Active ingredients of Huidouba in the treatment of type 2 diabetes mellitus. *J Chin Med Mater* 36(08)**,** 1313-1316.

Xi, X.F., Cui, J.R., and Wang, Y. (2009). Effects of *Phyllanthus emblica* L. Extract on Expression of Peroxisome Prolifemtor-activated Receptor γ (PPARγ) of Insulin Resistance Rats. *Food Sci* 30(05)**,** 253-256. doi: 10.7506/spkx1002-6630-200905058.

Xu, T., Ge, Y., Du, H., Li, Q., Xu, X., Yi, H., et al. (2021). Berberis kansuensis extract alleviates type 2 diabetes in rats by regulating gut microbiota composition. *J Ethnopharmacol* 273**,** 113995. doi: 10.1016/j.jep.2021.113995.

Yang, K., Bai, Y., Yu, N., Lu, B., Han, G., Yin, C., et al. (2020). Huidouba Improved Podocyte Injury by Down-Regulating Nox4 Expression in Rats With Diabetic Nephropathy. *Front Pharmacol* 11**,** 587995. doi: 10.3389/fphar.2020.587995.

Yang, M.H., Vasquez, Y., Ali, Z., Khan, I.A., and Khan, S.I. (2013). Constituents from Terminalia species increase PPARα and PPARγ levels and stimulate glucose uptake without enhancing adipocyte differentiation. *J Ethnopharmacol* 149(2)**,** 490-498. doi: 10.1016/j.jep.2013.07.003.

Ye, F., Yue, L.J., Fan, G., Meng, X.L., and Lai, G.R. (2016). Effects of water extract from cortex of *Berberi dictyophylla* on diabetic retinopathy in spontaneous Type 2 diabetic *db/db* Mice (Ⅰ). *Chin J Exp Tradit Med Form* 22(02)**,** 82-86. doi: 10.13422/j.cnki.syfjx.2016020082.

Zhang, Y., Yan, L.S., Ding, Y., Cheng, B.C.Y., Luo, G., Kong, J., et al. (2020a). Edgeworthia gardneri (Wall.) Meisn. Water Extract Ameliorates Palmitate Induced Insulin Resistance by Regulating IRS1/GSK3β/FoxO1 Signaling Pathway in Human HepG2 Hepatocytes. *Front Pharmaco* 10**,** 1666. doi: 10.3389/fphar.2019.01666.

Zhang, Y.J., Xiang, R.Q., Fang, S.D., Huang, K., Fan, Y., and Liu, T. (2020b). Experimental Study on the Effect of Tibetan Medicine Triphala on the Proliferation and Apoptosis of Pancreatic Islet β Cells through Incretin–cAMP Signaling Pathway. *Biol Pharm Bull* 43(2)**,** 289-295. doi: 10.1248/bpb.b19-00562.

Zhang, Z.W., Xu, H.Y., Zhao, H., Geng, Y., Ren, Y.L., Guo, L., et al. (2019). *Edgeworthia gardneri* (Wall.) Meisn. water extract improves diabetes and modulates gut microbiota. *J Ethnopharmaco* 239**,** 111854. doi: 10.1016/j.jep.2019.111854.

Zhao, D.G., Zhou, A.Y., Du, Z.Y., Zhang, Y., Zhang, K., and Ma, Y.Y. (2015). Coumarins with α-glucosidase and α-amylase inhibitory activities from the flower of Edgeworthia gardneri. *Fitoterapia* 107**,** 122-127. doi: 10.1016/j.fitote.2015.10.012.

Zhou, B.H., Ye, F., Yue, L.J., Meng, X.L., Fan, G., and Lai, X.R. (2016). Effects of water extract from cortex of *Berberi dictyophylla* on diabetic retinopathy in spontaneous Type 2 diabetic *db/db* Mice (Ⅱ). *Chin J Exp Tradit Med Form* 22(24)**,** 116-121. doi: 10.13422/j.cnki.syfjx.2016240116.

Zhou, J., Zhang, C., Zheng, G.H., and Qiu, Z. (2018a). Emblic Leafflower (*Phyllanthus emblica* L.) Fruits Ameliorate Vascular Smooth Muscle Cell Dysfunction in Hyperglycemia: An Underlying Mechanism Involved in Ellagitannin Metabolite Urolithin A. *Evid Based Complement Alternat Med* 2018**,** 8478943. doi: 10.1155/2018/8478943.

Zhou, Y.L., Jia, X.B., Liu, J.P., and Feng, L. (2018b). Protection of Huidouba, a Tibetan medicine on renal injury in STZ-induced type Ⅱ diabetic mice. *Chin Tradi Pat Med* 40(03)**,** 505-511. doi: 10.3969/j.issn.1001-1528.2018.03.001.

Zhuang, M., Qiu, H., Li, P., Hu, L., Wang, Y., and Rao, L. (2018). Islet protection and amelioration of type 2 diabetes mellitus by treatment with quercetin from the flowers of Edgeworthia gardneri. *Drug Des Devel Ther* 12**,** 955-966. doi: 10.2147/dddt.S153898.

Zuo, X.S., Ma, D.Q., Fang, S.D., Hua, J., Fan, Y., and Zhang, Y. (2018). Inhibition of High Glucose-induced Apoptosis of Pancreatic β-cells by Gallic Acid in *Phyllanthus Emblica*. *J Kunming Med Univ* 39(06)**,** 14-21.

**195 collected DM-TMM-related research articles：**

Ahmed, S., Ding, X., and Sharma, A. (2021). Exploring scientific validation of Triphala Rasayana in ayurveda as a source of rejuvenation for contemporary healthcare: An update. *J Ethnopharmacol* 273**,** 113829. doi: 10.1016/j.jep.2021.113829.

Ai, X.P., Hou, Y., Wang, X.B., Wang, X.Y., Liang, Y.S., Zhu, Z.W., et al. (2019a). Amelioration of dry eye syndrome in db/db mice with diabetes mellitus by treatment with Tibetan Medicine Formula Jikan Mingmu Drops. *J Ethnopharmacol* 241**,** 111992. doi: 10.1016/j.jep.2019.111992.

Ai, X.P., Wang, X.B., Wang, X.Y., Hou, Y., Liang, Y.S., Huang, W.Y., et al. (2019b). Evaluation on the protection mechanism of *Berberidis dictyophyllae* Cortex on

STZ-induced diabetic nephropathy in rats based on metabolomics. *Pharmacol Clin Chin Mater Med* 35(02)**,** 67-73. doi: 10.13412/j.cnki.zyyl.2019.02.015.

Akarshini, A.M., and Aruna (2014). Management of Madhumeha Janya Upadrava with special reference to diabetic nephropathy - A clinical study. *Ayu* 35(4)**,** 378-383. doi: 10.4103/0974-8520.158987.

Ansari, A., Shahriar, M.S., Hassan, M.M., Das, S.R., Rokeya, B., Haque, M.A., et al. (2014). Emblica officinalis improves glycemic status and oxidative stress in STZ induced type 2 diabetic model rats. *Asian Pac J Trop Med* 7(1)**,** 21-25. doi: 10.1016/s1995-7645(13)60185-6.

Ayyanar, M., and Ignacimuthu, S. (2011). Ethnobotanical survey of medicinal plants commonly used by Kani tribals in Tirunelveli hills of Western Ghats, India. *J Ethnopharmacol* 134(3)**,** 851-864. doi: 10.1016/j.jep.2011.01.029.

Bai, Y.H., Lu, B.N., and Pang, Z.R. (2016). Development of The Five Medicinal and Edible Uyghur Medicine on Treating Type 2 Diabetes Mellitus. *J Tianjin Univ Tradit Chin Med* 35(06)**,** 428-432. doi: 10.11656/j.issn.1673-9043.2016.06.17.

Bai, Y.H., Shi, D.X., Lu, H.Y., Yang, K.B., Zhao, H.H., Lu, B.N., et al. (2021). Hypoglycemic effects of Tibetan medicine Huidouba in STZ-induced diabetic mice and db/db mice. *Chin Herb Med* 13(2)**,** 202-209. doi: 10.1016/j.chmed.2021.02.001.

Balusamy, S.R., Veerappan, K., Ranjan, A., Kim, Y.J., Chellappan, D.K., Dua, K., et al. (2020). Phyllanthus emblica fruit extract attenuates lipid metabolism in 3T3-L1 adipocytes via activating apoptosis mediated cell death. *Phytomedicine* 66**,** 153129. doi: 10.1016/j.phymed.2019.153129.

Bansode, T.S., Salalkar, B.K., Dighe, P., Nirmal, S., and Dighe, S. (2017). Comparative evaluation of antidiabetic potential of partially purified bioactive fractions from four medicinal plants in alloxan-induced diabetic rats. *Ayu* 38(3-4)**,** 165-170. doi: 10.4103/ayu.AYU_18_17.

Bao, Y.R., Yang, Y., Xu, D.D., Xie, S.X., Liu, Y., and Xu, Y.J. (2019). Research progress in chemical constituents and pharmacological activities of *Edgeworthia gardneri*. *China Pharm* 30(02)**,** 277-281. doi: 10.6039/j.issn.1001-0408.2019.02.28.

Berdja, S., Smail, L., Saka, B., Neggazi, S., Haffaf el, M., Benazzoug, Y., et al. (2016). Glucotoxicity Induced Oxidative Stress and Inflammation *In vivo* and *In vitro* in Psammomys obesus: Involvement of Aqueous Extract of Brassica rapa rapifera. *Evid Based Complement Alternat Med* 2016**,** 3689208. doi: 10.1155/2016/3689208.

Bhatia, H., Sharma, Y.P., Manhas, R.K., and Kumar, K. (2014). Ethnomedicinal plants used by the villagers of district Udhampur, J&K, India. *J Ethnopharmacol* 151(2)**,** 1005-1018. doi: 10.1016/j.jep.2013.12.017.

Cai, W.L., Han, M.M., Sun, Y.M., Li, Z.M., and Peng, L. (2019). Study on the Chemical Constituents and Inhibitory Activity against α-Glucosidase of Ethyl Acetate Extract from Huidouba. *J Jiangxi Sci Techno Normal Univ* (06)**,** 60-63.

Chan, X.C., and Zhan, R.J. (2019). Protective Effect of Edgeworthia Gardneri Flos on Pancreas in Diabetes Mellitus Model Rats Induced by Tacrolimus and Its Mechanism. *Chin J Mod Appl Pharm* 36(16)**,** 2008-2013.

Chandel, H.S., Pathak, A.K., and Tailang, M. (2011). Standardization of some herbal antidiabetic drugs in polyherbal formulation. *Pharmacognosy Res* 3(1)**,** 49-56. doi: 10.4103/0974-8490.79116.

Chang, X.J., Ma, R.L., and Wu, Q. (2021). A preliminary study on the effect of Shibawei Heziliniao pills on gastroparesis rats with type 2 diabetes mellitus. *Chin J Gerontol* 41(03)**,** 604-606. doi: 10.3969/j.issn.1005-9202.2021.03.044.

Chen, C.X., Xie, M.N., and Huang, Z.H. (2018a). Polysaccharides from Huidouba and Its Effect on Type-2 Diabetes Mellitus. *Agric Biotechnol* 7(02)**,** 156-160. doi: 10.19759/j.cnki.2164-4993.2018.02.037.

Chen, J., Li, L., Zhou, X., Li, B., Zhang, X., and Hui, R. (2018b). Structural characterization and α-glucosidase inhibitory activity of polysaccharides extracted from Chinese traditional medicine Huidouba. *Int J Biol Macromol* 117**,** 815-819. doi: 10.1016/j.ijbiomac.2018.05.192.

Chen, J.C., Li, L., Zhou, X., Sun, P.Y., Li, B., and Zhang, X. (2018c). Preliminary characterization and antioxidant and hypoglycemic activities *in vivo* of polysaccharides from Huidouba. *Food Func* 9(12)**,** 6337-6348. doi: 10.1039/c8fo01117f.

Chen, T.S., Liou, S.Y., Wu, H.C., Tsai, F.J., Tsai, C.H., Huang, C.Y., et al. (2011). Efficacy of epigallocatechin-3-gallate and Amla (Emblica officinalis) extract for the treatment of diabetic-uremic patients. *J Med Food* 14(7-8)**,** 718-723. doi: 10.1089/jmf.2010.1195.

Chen , W.B., Shi, Y., Zhang, B.W., and Ye, Y.H. (2019). Ｒesearch Progress on Chemical Constituents and Pharmacological Activity of Flavonoids from *Brassica Rapa*. *J Jiangxi Univ Tradit Chin Med* 31(03)**,** 115-118.

Chen, X.H., Liu, Y., Weng, Y.X., Kang, W.J., and Yang, S.B. (2014a). Hypoglycemic mechanism of volatile oil of plateau plant *Brassica rapa* L．in hyperlipidemic and hyperglycemic mice. *J Shandong Univ* (*Health Sci*) 52(12)**,** 20-23. doi: 10.6040/j.issn.1671-7554.0.2014.172.

Chen, X.H., Liu, Y., Weng, Y.X., Kang, W.J., and Yang, S.B. (2014b). Study on Hypoglycemic Action of Plateau *Brassica rapa* Volatile Oil Components. *Chin J Exp Tradit Med Form* 20(15)**,** 131-133. doi: 10.13422/j.cnki.syfjx.2014150131.

Chen, X.H., Wen, S.D., Wu, P., Sa, Y.P., and Ren, S.C. (2013). Study on Hypoglycemic Effect of Different Extracts of *Brassica rapa* on Diabetic Model Mice. *China Pharm* 24(07)**,** 596-598. doi: 10.6039/j.issn.1001-0408.2013.07.07.

Cheng, Y., Song, J.H., Yang, S.R., and J., L.S. (2013). Exploring hypoglycemic by alloxan-induced diabetic mice model with Gray pocket extraction. *Lishizhen Med Mater Med Res* 24(04)**,** 852-854. doi: 10.3969/j.issn.1008-0805.2013.04.036.

Cock, I.E. (2015). The medicinal properties and phytochemistry of plants of the genus Terminalia (Combretaceae). *Inflammopharmacology* 23(5)**,** 203-229. doi: 10.1007/s10787-015-0246-z.

D'Souza J, J., D'Souza P, P., Fazal, F., Kumar, A., Bhat, H.P., and Baliga, M.S. (2014). Anti-diabetic effects of the Indian indigenous fruit Emblica officinalis Gaertn: active constituents and modes of action. *Food Funct* 5(4)**,** 635-644. doi: 10.1039/c3fo60366k.

Dan, B., Li, L.Y., Li, Y.H., and Chen, X.H. (2020). Effect of Huidouba, a Tibetan medcine on TGF-β1/Smads signal pathway in diabetic nephropathy rats. *Northwest Pharm J* 35(04)**,** 531-534. doi: 10.3969/j.issn.1004-2407.2020.04.12.

Das, N.D., Jung, K.H., Park, J.H., Choi, M.R., Lee, H.T., Kim, M.S., et al. (2012). Proteomic analysis of Terminalia chebula extract-dependent changes in human lymphoblastic T cell protein expression. *J Med Food* 15(7)**,** 651-657. doi: 10.1089/jmf.2011.1998.

Das, N.D., Jung, K.H., Park, J.H., Mondol, M.A., Shin, H.J., Lee, H.S., et al. (2011). Terminalia chebula extract acts as a potential NF-κB inhibitor in human lymphoblastic T cells. *Phytother Res* 25(6)**,** 927-934. doi: 10.1002/ptr.3398.

Deng, R. (2012). A review of the hypoglycemic effects of five commonly used herbal food supplements. *Recent Pat Food Nutr Agric* 4(1)**,** 50-60. doi: 10.2174/2212798411204010050.

Dro, J., Tashi, D.Z., Tashi, T., and Liao, X.X. (2019). The Effect of *Phyllanthus Emblica* Compound Pulvis on Blood Glucose and Hemorheology of Rats with Diabetes *Guangdong Chem Ind* 46(07)**,** 54-55.

Du, H., Li, Q., Yi, H., Xu, T., Xu, X.M., Kuang, T.T., et al. (2020). Anti-Diabetic Effects of Berberis kansuensis Extract on Type 2 Diabetic Rats Revealed by (1) H-NMR-Based Metabolomics and Biochemistry Analysis. *Chem Biodivers* 17(10)**,** e2000413. doi: 10.1002/cbdv.202000413.

Duan, B.L., Zhao, Z.Q., Liao, W.F., Xiong, H., Liu, S.S., Yin, L., et al. (2017a). Antidiabetic Effect of Tibetan Medicine Tang-Kang-Fu-San in db/db Mice via Activation of PI3K/Akt and AMPK Pathways. *Front Pharmacol* 8. doi: 10.3389/fphar.2017.00535.

Duan, B.L., Zhao, Z.Q., Lin, L., Jin, J., Zhang, L.J., Xiong, H., et al. (2017b). Antidiabetic Effect of Tibetan Medicine Tang-Kang-Fu-San on High-Fat Diet and Streptozotocin-Induced Type 2 Diabetic Rats. *Evid Based Complement Alternat Med* 2017(7302965)**,** 1-9. doi: 10.1155/2017/7302965.

Faizal, P., Suresh, S., Satheesh Kumar, R., and Augusti, K.T. (2009). A study on the hypoglycemic and hypolipidemic effects of an ayurvedic drug Rajanyamalakadi in diabetic patients. *Indian J Clin Biochem* 24(1)**,** 82-87. doi: 10.1007/s12291-009-0014-1.

Fan, G., Li, Q., Xu, X.M., Du, H., Xue, T., Lai, X.R., et al. (2020). Quality evaluation of different Berberidis Cortex species based on 1H-NMR metabolomics and anti-diabetic activity. *China J Chin Mater Med***,** 1-11. doi: 10.19540/j.cnki.cjcmm.20200620.201.

Fatima, N., Hafizur, R.M., Hameed, A., Ahmed, S., Nisar, M., and Kabir, N. (2017). Ellagic acid in Emblica officinalis exerts anti-diabetic activity through the action on β-cells of pancreas. *Eur J Nutr* 56(2)**,** 591-601. doi: 10.1007/s00394-015-1103-y.

Fatima, N., Pingali, U., and Muralidhar, N. (2014). Study of pharmacodynamic interaction of Phyllanthus emblica extract with clopidogrel and ecosprin in patients with type II diabetes mellitus. *Phytomedicine* 21(5)**,** 579-585. doi: 10.1016/j.phymed.2013.10.024.

Feng, G.R. (2003). Therapeutic effect of *Lamiophlomis rotata* on diabetic vitreous hemorrhage. *Guangxi Med J* (04)**,** 619-620.

Ganeshpurkar, A., Jain, S., and Agarwal, S. (2015). Experimental studies on glycolytic enzyme inhibitory and antiglycation potential of Triphala. *Ayu* 36(1)**,** 96-100. doi: 10.4103/0974-8520.169000.

Gao, D., Zhang, Y.L., Xu, P., Lin, Y.X., Yang, F.Q., Liu, J.H., et al. (2015). *In vitro* evaluation of dual agonists for PPARγ/β from the flower of Edgeworthia gardneri (wall.) Meisn. *J Ethnopharmacol* 162**,** 14-19. doi: 10.1016/j.jep.2014.12.034.

Gao, H., Huang, Y.N., Gao, B., and Kawabata, J. (2008). Chebulagic acid is a potent alpha-glucosidase inhibitor. *Biosci Biotechnol Biochem* 72(2)**,** 601-603. doi: 10.1271/bbb.70591.

Geng, Y., Yang, H.M., Xu, H.Y., and Shi, J.-S. (2013). α-Glucosidase inhibitory activity of the alabastrum of Edgeworthia gardneri (Wall.) Meissn. *J Food Sci Biotechnol* 32**,** 967-971.

Gujarathi, R.A., Dwivedi, R., and Vyas, M.K. (2014). An observational pilot study on the effect of Gomutra Haritaki, diet control and exercise in the management of Sthaulya (obesity). *Ayu* 35(2)**,** 129-134. doi: 10.4103/0974-8520.146206.

Guo, Z.Y., Huang, Y.X., and Wang, G.Q. (2014). Advances in Research of Emblica Fruit (*Phyllanthus Emblica* L.) for the Prevention and Treatment of Diabetes Mellitus and Complications. *Strait Pharm J* 26(12)**,** 1-4.

Gupta, A., Agarwal, N.K., and Byadgi, P.S. (2014). Clinical assessment of dietary interventions and lifestyle modifications in Madhumeha (type- 2 Diabetes Mellitus). *Ayu* 35(4)**,** 391-397. doi: 10.4103/0974-8520.158997.

Haierguli, M., Zulipiyan, A., and Hailiqian, T. (2020). Preliminary study on the hypoglycemic effect of neutral polysaccharide from

*Brassica rapa* L. *J Food Saf Qual* 11(02)**,** 387-392. doi: 10.19812/j.cnki.jfsq11-5956/ts.2020.02.010.

Han, G., Yuan, H.Z., Dong, Y., Zhai, G.Y., and Fan, Y. (2009). Blood Glucose-reducing Effects of Phyllanthi Fructus Fruit Extracts on Diabetic Mice. *Food Sci* 30(09)**,** 210-212.

Hassanpour Fard, M., Naseh, G., Lotfi, N., Hosseini, S.M., and Hosseini, M. (2015). Effects of aqueous extract of turnip leaf (Brassica rapa) in alloxan-induced diabetic rats. *Avicenna J Phytomed* 5(2)**,** 148-156.

Hiraganahalli, B.D., Chinampudur, V.C., Dethe, S., Mundkinajeddu, D., Pandre, M.K., Balachandran, J., et al. (2012). Hepatoprotective and antioxidant activity of standardized herbal extracts. *Pharmacogn Mag* 8(30)**,** 116-123. doi: 10.4103/0973-1296.96553.

Hu, W. (2012). Effects of Phyllanthus Emblica extracts on the insulin signal pathway in muscle and fat tissues of diabetic rats *Chin J Tissue Eng Res* 16(11)**,** 2007-2010.

Hua, B.J., and Yang, Z.Z. (2019). The Clinical Value of Tibetan Medicine Shibawei Hezi Liniao Wan in the Treatment of Diabetes. *J Clin Med* 6(07)**,** 149. doi: 10.16281/j.cnki.jocml.2019.07.130.

Huang, H.Z., Qiu, M., Lin, J.Z., Li, M.Q., Ma, X.T., Ran, F., et al. (2021). Potential effect of tropical fruits Phyllanthus emblica L. for the prevention and management of type 2 diabetic complications: a systematic review of recent advances. *Eur J Nutr*. doi: 10.1007/s00394-020-02471-2.

Huang, S., Liu, Z., Liu, H., Lee, D., Wang, J., Yuan, R., et al. (2019). Nepeta angustifolia attenuates responses to vascular inflammation in high glucose-induced human umbilical vein endothelial cells through heme oxygenase-1 induction. *J Ethnopharmacol* 231**,** 187-196. doi: 10.1016/j.jep.2018.11.015.

Huang, S., Tan, M., Guo, F., Dong, L., Liu, Z., Yuan, R., et al. (2020). Nepeta angustifolia C. Y. Wu improves renal injury in HFD/STZ-induced diabetic nephropathy and inhibits oxidative stress-induced apoptosis of mesangial cells. *J Ethnopharmacol* 255**,** 112771. doi: 10.1016/j.jep.2020.112771.

Huang, Y.N., Zhao, D.D., Gao, B., Zhong, K., Zhu, R.X., Zhang, Y., et al. (2012). Anti-hyperglycemic effect of chebulagic acid from the fruits of Terminalia chebula Retz. *Int J Mol Sci* 13(5)**,** 6320-6333. doi: 10.3390/ijms13056320.

Ishtiaq, M., Maqbool, M., Ajaib, M., Ahmed, M., Hussain, I., Khanam, H., et al. (2021). Ethnomedicinal and folklore inventory of wild plants used by rural communities of valley Samahni, District Bhimber Azad Jammu and Kashmir, Pakistan. *PLoS One* 16(1)**,** e0243151. doi: 10.1371/journal.pone.0243151.

Jokar, A., Masoomi, F., Sadeghpour, O., Nassiri-Toosi, M., and Hamedi, S. (2016). Potential therapeutic applications for Terminalia chebula in Iranian traditional medicine. *J Tradit Chin Med* 36(2)**,** 250-254. doi: 10.1016/s0254-6272(16)30035-8.

Kalekar, S.A., Munshi, R.P., and Thatte, U.M. (2013). Do plants mediate their anti-diabetic effects through anti-oxidant and anti-apoptotic actions? an *in vitro* assay of 3 Indian medicinal plants. *BMC Complement Altern Med* 13**,** 257. doi: 10.1186/1472-6882-13-257.

Kang, W.J., Zhang, G.M., Zhao, X.H., Tong, L., Wang, J.H., and Liu, Z.H. (2011). Hypoglycemic Effects of Different Solvent Extracts from Phmllanthi fructus. *J Anhui Agri Sci* 39(30)**,** 18545-18547. doi: 10.13989/j.cnki.0517-6611.2011.30.123.

Kim, M.S., Lee, D.Y., Lee, J., Kim, H.W., Sung, S.H., Han, J.S., et al. (2018). Terminalia chebula extract prevents scopolamine-induced amnesia via cholinergic modulation and anti-oxidative effects in mice. *BMC Complement Altern Med* 18(1)**,** 136. doi: 10.1186/s12906-018-2212-y.

Kumar, N.P., Annamalai, A.R., and Thakur, R.S. (2009). Antinociceptive property of Emblica officinalis Gaertn (Amla) in high fat diet-fed/low dose streptozotocin induced diabetic neuropathy in rats. *Indian J Exp Biol* 47(9)**,** 737-742.

Kurian, G.A., Manjusha, V., Nair, S.S., Varghese, T., and Padikkala, J. (2014). Short-term effect of G-400, polyherbal formulation in the management of hyperglycemia and hyperlipidemia conditions in patients with type 2 diabetes mellitus. *Nutrition* 30(10)**,** 1158-1164. doi: 10.1016/j.nut.2014.02.026.

Kusirisin, W., Srichairatanakool, S., Lerttrakarnnon, P., Lailerd, N., Suttajit, M., Jaikang, C., et al. (2009). Antioxidative activity, polyphenolic content and anti-glycation effect of some Thai medicinal plants traditionally used in diabetic patients. *Med Chem* 5(2)**,** 139-147. doi: 10.2174/157340609787582918.

Lai, X., Tong, D., Ai, X., Wu, J., Luo, Y., Zuo, F., et al. (2018). Amelioration of diabetic nephropathy in db/db mice treated with tibetan medicine formula Siwei Jianghuang Decoction Powder extract. *Sci Rep* 8(1)**,** 16707. doi: 10.1038/s41598-018-35148-2.

Lamaala (2017). The Clinical Value of Tibetan Medicine Shibawei Hezi Liniao Wan in the Treatment of Diabetes. *Smart Healthcare* 3(19)**,** 40-41. doi: 10.19335/j.cnki.2096-1219.2017.19.17.

Lee, H.H., Paudel, K.R., and Kim, D.W. (2015). Terminalia chebula Fructus Inhibits Migration and Proliferation of Vascular Smooth Muscle Cells and Production of Inflammatory Mediators in RAW 264.7. *Evid Based Complement Alternat Med* 2015**,** 502182. doi: 10.1155/2015/502182.

Li, D., Peng, C., Xie, X., Mao, Y., Li, M., Cao, Z., et al. (2014). Antidiabetic effect of flavonoids from Malus toringoides (Rehd.) Hughes leaves in diabetic mice and rats. *J Ethnopharmacol* 153(3)**,** 561-567. doi: 10.1016/j.jep.2014.02.026.

Li, D., Peng, C., Xie, X.F., Mao, Y., Li, M., and Fan, D., Q. (2013a). Effects of Three Different Extracts from *Malus toringoides* in Experimental Diabetic Mice. *Chin J Exp Tradit Med Form* 19(21)**,** 199-203. doi: 10.11653/syfj2013210199.

Li, M., Ding, L., Hu, Y.L., Qin, L.L., Wu, Y., Liu, W., et al. (2021). Herbal formula LLKL ameliorates hyperglycaemia, modulates the gut microbiota and regulates the gut-liver axis in Zucker diabetic fatty rats. *J Cell Mol Med* 25(1)**,** 367-382. doi: 10.1111/jcmm.16084.

Li, M., Nan, C.Y., Zhu, J.X., and Zhong, G.C. (2018). Screening of anti-T2DM PPARs agonist from Lvluohua. *Chin Tradit Pat Med* 40(10)**,** 2285-2288. doi: 10.3969 /j.issn.1001-1528.2018.10.034.

Li, M., Wu, L.L., Qin, L.L., Hu, Y.L., Qin, T.Y., Ding, L., et al. (2020). Research Progress on Medicinal Effects of Flower of *Edgeworthia Gardneri*. *Chin J Exp Tradit Med Form* 26(06)**,** 209-215. doi: 10.13422/j.cnki.syfjx.20192125.

Li, Y., Xie, X.F., Li, D., Zhuo, X., Tang, D.Q., Li, R.Y., et al. (2013b). Pharmacological Effects of Total Flavonoids from E’se on Diabetes Mellitus. *China Foreign Med Treat* 32(14)**,** 25-27. doi: 10.16662/j.cnki.1674-0742.2013.14.006.

Li, Z.M., and Peng, L. (2012). Inhibitory Effect on α-Glucosidase of Huidouba Extracts *in vitro*. *Lishizhen Med Mater Med Res* 23(06)**,** 1379-1380. doi: 10.3969/j.issn.1008-0805.2012.06.026.

Li, Z.M., Sun, Y.M., Peng, L., Liu, Y., and Du, H. (2015). The inhibitory effects of compatibility of different Huidouba etracts on α-glucosidase. *Chin Tradit Pat Med* 37(04)**,** 879-882. doi: 10.3969/j.issn.1001-1528.2015.04. 043.

Liang, P.Y., Liang, L.L., and Wu, Q. (2019). The protective effect of eighteen flavor *myrobalan* diuretic pills on diabetic rats. *Chin J Gerontol* 39(01)**,** 141-144. doi: 10.3969/j.issn.1005-9202.2019.01.050.

Liang, P.Y., Yin, L.J., Hua, Q.S., and Wu, Q. (2016). Effect of Shibawei Keziliniao pills on blood glucose of rats with plateau diabetes. *J Baotou Med Coll* 32(12)**,** 3-5. doi: 10.16833/j.cnki.jbmc.2016.12.002.

Lin, Y.X., and Xia, Z.N. (2016). Screening Active Fractions of Peroxisome Proliferator Activated Receptor γ in Natural Medicines with Potential Antidiabetic Activity. *Nat Prod Res Dev* 28(04)**,** 505-513.

Liu, F., Qin, H.F., and Liu, S.Q. (2012). Research progress on the chemical constituents and pharmacological activities of Chebulae Fructus. *Chin Pharm* 23(07)**,** 670-672. doi: 10.6039/j.issn.1001-0408.2012.07.35.

Liu, Y., Chen, Z.J., Liang, Y.T., Cui, L.Y., ZHeng, D., Guo, W.L., et al. (2009a). Extraction and Anti-diabetic Activity on Alloxan-induced Diabetic Mice of Polysaccharides from Huidouba. *Chem Res Chin Univ* 25(05)**,** 681-685.

Liu, Y., Lu, J.H., Zheng, D., Meng, Q.F., Guo, W.L., and Teng, L.R. (2009b). Optimization of ultrasonic assisted extraction of Huidouba polysaccharides

and its hypoglycemic effect on alloxan-diabetic mice. *J Jilin Univ* (*Eng Technol Edit*) 39(S1)**,** 360-365. doi: 10.13229/j.cnki.jdxbgxb2009.s1.045.

Luo, P., Liu, Y., Tan, Z.H., and Zhang, Z.F. (2013). Study on antidiabetic constituents of Tibetan Medicine Meiduoluomi. *Chin Tradit Herb Drugs* 44(14)**,** 1962-1966. doi: 10.7501/j.issn.0253-2670.2013.14.018.

Luo, P., Tan, Z.H., Zhang, Z.F., Zhang, H., Liu, X.F., and Mo, Z.J. (2008). Scutellarin isolated from Erigeron multiradiatus inhibits high glucose-mediated vascular inflammation. *Yakugaku Zasshi* 128(9)**,** 1293-1299. doi: 10.1248/yakushi.128.1293.

Ma, Y.Y., Zhao, D.G., Zhou, A.Y., Zhang, Y., Du, Z., and Zhang, K. (2015). α-Glucosidase Inhibition and Antihyperglycemic Activity of Phenolics from the Flowers of Edgeworthia gardneri. *J Agric Food Chem* 63(37)**,** 8162-8169. doi: 10.1021/acs.jafc.5b03081.

Majeed, M., Majeed, S., Mundkur, L., Nagabhushanam, K., Arumugam, S., Beede, K., et al. (2020). Standardized Emblica officinalis fruit extract inhibited the activities of α-amylase, α-glucosidase, and dipeptidyl peptidase-4 and displayed antioxidant potential. *J Sci Food Agric* 100(2)**,** 509-516. doi: 10.1002/jsfa.10020.

Marwat, S.K., Rehman, F., Khan, E.A., Khakwani, A.A., Ullah, I., Khan, K.U., et al. (2014). Useful ethnophytomedicinal recipes of angiosperms used against diabetes in South East Asian Countries (India, Pakistan & Sri Lanka). *Pak J Pharm Sci* 27(5)**,** 1333-1358.

Mathiyazhagan, J., and Kodiveri Muthukaliannan, G. (2020). The role of mTOR and oral intervention of combined Zingiber officinale-Terminalia chebula extract in type 2 diabetes rat models. *J Food Biochem* 44(7)**,** e13250. doi: 10.1111/jfbc.13250.

Meng, F.X., Wang, L.Y., Song, J.J., Zheng, D., and Teng, L.R. (2012). Study on the hypoglycemic effects of Huidouba polysaccharides in diabetic mice. *Lishizhen Med Mater Med Res* 23(06)**,** 1557-1558. doi: 10.3969/j.issn.1008-0805.2012.06.107.

Meng, Z.M., Geng, Y., Li, H., Xu, H.Y., Zhao, H., Liu, M., et al. (2019). Ameliorative Effects of Fraction 1 from the Flower of *Edgeworthia gardneri* (Wall.) Meisn on Insulin Resistance of C2C12 Cells. *J Food Sci Biotechnol* 38(11)**,** 55-62. doi: 10.3969/j.issn.1673-1689.2019.11.008.

Moïse, M.M., Benjamin, L.M., Doris, T.M., Dalida, K.N., and Augustin, N.O. (2012). Role of Mediterranean diet, tropical vegetables rich in antioxidants, and sunlight exposure in blindness, cataract and glaucoma among African type 2 diabetics. *Int J Ophthalmol* 5(2)**,** 231-237. doi: 10.3980/j.issn.2222-3959.2012.02.23.

Murali, Y.K., Anand, P., Tandon, V., Singh, R., Chandra, R., and Murthy, P.S. (2007). Long-term effects of Terminalia chebula Retz. on hyperglycemia and associated hyperlipidemia, tissue glycogen content and *in vitro* release of insulin in streptozotocin induced diabetic rats. *Exp Clin Endocrinol Diabetes* 115(10)**,** 641-646. doi: 10.1055/s-2007-982500.

Murali, Y.K., Chandra, R., and Murthy, P.S. (2004). Antihyperglycemic effect of water extract of dry fruits ofTerminalia chebula in experimental diabetes mellitus. *Indian J Clin Biochem* 19(2)**,** 202-204. doi: 10.1007/bf02894285.

Nain, P., Saini, V., Sharma, S., and Nain, J. (2012). Antidiabetic and antioxidant potential of Emblica officinalis Gaertn. leaves extract in streptozotocin-induced type-2 diabetes mellitus (T2DM) rats. *J Ethnopharmacol* 142(1)**,** 65-71. doi: 10.1016/j.jep.2012.04.014.

Nalamolu, K.R., and Nammi, S. (2006). Antidiabetic and renoprotective effects of the chloroform extract of Terminalia chebula Retz. seeds in streptozotocin-induced diabetic rats. *BMC Complement Altern Med* 6**,** 17. doi: 10.1186/1472-6882-6-17.

Nampoothiri, S.V., Prathapan, A., Cherian, O.L., Raghu, K.G., Venugopalan, V.V., and Sundaresan, A. (2011). *In vitro* antioxidant and inhibitory potential of Terminalia bellerica and Emblica officinalis fruits against LDL oxidation and key enzymes linked to type 2 diabetes. *Food Chem Toxicol* 49(1)**,** 125-131. doi: 10.1016/j.fct.2010.10.006.

Nan, C.Y., Lu, Y.X., Zhu, J.X., Jiang, W., Zhong, G.Y., and Li, M. (2018). Research progress on chemical constituents and pharmacological activities of Edgeworthia plants. *Chin Tradit Pat Med* 40(01)**,** 166-171.

Nan, C.Y., Zhong, G.Y., Zhu, J.X., Jiang, W., and Li, M. (2019). Effects of Components from Edgeworthia gardneri Meissn on PPARs Level in 3T3-L1 Cells. *Tradit Chin Drug Res Pharmaco* 30(03)**,** 282-288. doi: 10.19378/j.issn.1003-9783.2019.03.003.

Ocvirk, S., Kistler, M., Khan, S., Talukder, S.H., and Hauner, H. (2013). Traditional medicinal plants used for the treatment of diabetes in rural and urban areas of Dhaka, Bangladesh--an ethnobotanical survey. *J Ethnobiol Ethnomed* 9**,** 43. doi: 10.1186/1746-4269-9-43.

Okada, Y., Okada, M., and Sagesaka, Y. (2010). Screening of dried plant seed extracts for adiponectin production activity and tumor necrosis factor-alpha inhibitory activity on 3T3-L1 adipocytes. *Plant Foods Hum Nutr* 65(3)**,** 225-232. doi: 10.1007/s11130-010-0184-2.

P, S., Zinjarde, S.S., Bhargava, S.Y., and Kumar, A.R. (2011). Potent α-amylase inhibitory activity of Indian Ayurvedic medicinal plants. *BMC Complement Altern Med* 11**,** 5. doi: 10.1186/1472-6882-11-5.

Panmei, R., Gajurel, P.R., and Singh, B. (2019). Ethnobotany of medicinal plants used by the Zeliangrong ethnic group of Manipur, northeast India. *J Ethnopharmacol* 235**,** 164-182. doi: 10.1016/j.jep.2019.02.009.

Patel, D.V., Chandola, H., Baghel, M.S., and Joshi, J.R. (2012). Clinical efficacy of Shankhapushpi and a herbo-mineral compound in type-II diabetes. *Ayu* 33(2)**,** 230-237. doi: 10.4103/0974-8520.105243.

Patel, S.S., and Goyal, R.K. (2011a). Cardioprotective effects of gallic acid in diabetes-induced myocardial dysfunction in rats. *Pharmacognosy Res* 3(4)**,** 239-245. doi: 10.4103/0974-8490.89743.

Patel, S.S., and Goyal, R.K. (2011b). Prevention of diabetes-induced myocardial dysfunction in rats using the juice of the Emblica officinalis fruit. *Exp Clin Cardiol* 16(3)**,** 87-91.

Patel, S.S., Goyal, R.K., Shah, R.S., Tirgar, P.R., and Jadav, P.D. (2013). Experimental study on effect of hydroalcoholic extract of Emblica officinalis fruits on glucose homeostasis and metabolic parameters. *Ayu* 34(4)**,** 440-444. doi: 10.4103/0974-8520.127731.

Patel, S.S., Shah, R.S., and Goyal, R.K. (2009). Antihyperglycemic, antihyperlipidemic and antioxidant effects of Dihar, a polyherbal ayurvedic formulation in streptozotocin induced diabetic rats. *Indian J Exp Biol* 47(7)**,** 564-570.

Peng, L., and Li, Z.M. (2010). Hypoglycemic effect of Huidouba on streptozocin-induced diabetic rats. *Lishizhen Med Mater Med Res* 21(12)**,** 3060-3061. doi: 10.3969/j.issn.1008-0805.2010.12.011.

Phimarn, W., Sungthong, B., and Itabe, H. (2021). Effects of Triphala on Lipid and Glucose Profiles and Anthropometric Parameters: A Systematic Review. *J Evid Based Integr Med* 26**,** 2515690x211011038. doi: 10.1177/2515690x211011038.

Pingali, U., Sukumaran, D., and Nutalapati, C. (2020). Effect of an aqueous extract of Terminalia chebula on endothelial dysfunction, systemic inflammation, and lipid profile in type 2 diabetes mellitus: A randomized double-blind, placebo-controlled clinical study. *Phytother Res* 34(12)**,** 3226-3235. doi: 10.1002/ptr.6771.

Puppala, M., Ponder, J., Suryanarayana, P., Reddy, G.B., Petrash, J.M., and LaBarbera, D.V. (2012). The isolation and characterization of β-glucogallin as a novel aldose reductase inhibitor from Emblica officinalis. *PLoS One* 7(4)**,** e31399. doi: 10.1371/journal.pone.0031399.

Rahmatullah, M., Azam, M.N., Khatun, Z., Seraj, S., Islam, F., Rahman, M.A., et al. (2012). Medicinal plants used for treatment of diabetes by the Marakh sect of the Garo tribe living in Mymensingh district, Bangladesh. *Afr J Tradit Complement Altern Med* 9(3)**,** 380-385. doi: 10.4314/ajtcam.v9i3.12.

Rajan, S.S., and Antony, S. (2008). Hypoglycemic effect of triphala on selected non insulin dependent Diabetes mellitus subjects. *Anc Sci Life* 27(3)**,** 45-49.

Rao, P.K., Hasan, S.S., Bhellum, B.L., and Manhas, R.K. (2015). Ethnomedicinal plants of Kathua district, J&K, India. *J Ethnopharmacol* 171**,** 12-27. doi: 10.1016/j.jep.2015.05.028.

Rao, T.P., Sakaguchi, N., Juneja, L.R., Wada, E., and Yokozawa, T. (2005). Amla (Emblica officinalis Gaertn.) extracts reduce oxidative stress in streptozotocin-induced diabetic rats. *J Med Food* 8(3)**,** 362-368. doi: 10.1089/jmf.2005.8.362.

Rathor, L., Pant, A., Awasthi, H., Mani, D., and Pandey, R. (2017). An antidiabetic polyherbal phytomedicine confers stress resistance and extends lifespan in Caenorhabditis elegans. *Biogerontology* 18(1)**,** 131-147. doi: 10.1007/s10522-016-9668-2.

Reddy, D.B., Reddy, T.C., Jyotsna, G., Sharan, S., Priya, N., Lakshmipathi, V., et al. (2009). Chebulagic acid, a COX-LOX dual inhibitor isolated from the fruits of Terminalia chebula Retz., induces apoptosis in COLO-205 cell line. *J Ethnopharmacol* 124(3)**,** 506-512. doi: 10.1016/j.jep.2009.05.022.

Ren, J.H., Suolang, O.Z., Shao, Y.L., De, Y., and Ding, Y.C. (2018). Investigation of Hypoglycemic Effect of Water-extraction from *Malus toringoides* Leaves in Diabetic Mice. *Food Res Dev* 39(21)**,** 27-32. doi: 10.3969/j.issn.1005-6521.2018.21.005.

Renzeng, J., C., W., Li, Q.E., Qieyang, R.Z., Pengmao, D.Z., and Gongque, J.Z. (2020). Mechanism prediction of therapeutic effect of Siwei-Jianghuangtang powder on diabetic nephropathy based on network pharmacology and RoNusZhurJes. *Chin Tradi Pat Med***,** 1-10.

Sasidharan, I., Sundaresan, A., Nisha, V.M., Kirishna, M.S., Raghu, K.G., and Jayamurthy, P. (2012). Inhibitory effect of Terminalia chebula Retz. fruit extracts on digestive enzyme related to diabetes and oxidative stress. *J Enzyme Inhib Med Chem* 27(4)**,** 578-586. doi: 10.3109/14756366.2011.603130.

Sharma, A., Tiwari, R.K., Sharma, V., Pandey, R.K., and Shukla, S.S. (2019). Antidiabetic Activity of an Ayurvedic Formulation Chaturmukha Rasa: A Mechanism Based Study. *J Pharmacopuncture* 22(2)**,** 115-121. doi: 10.3831/kpi.2019.22.015.

Sharma, P., Joshi, T., Joshi, T., Chandra, S., and Tamta, S. (2020). In silico screening of potential antidiabetic phytochemicals from Phyllanthus emblica against therapeutic targets of type 2 diabetes. *J Ethnopharmacol* 248**,** 112268. doi: 10.1016/j.jep.2019.112268.

Shokoohi, R., Kianbakht, S., Faramarzi, M., Rahmanian, M., Nabati, F., Mehrzadi, S., et al. (2017). Effects of an Herbal Combination on Glycemic Control and Lipid Profile in Diabetic Women: A Randomized, Double-Blind, Placebo-Controlled Clinical Trial. *J Evid Based Complementary Altern Med* 22(4)**,** 798-804. doi: 10.1177/2156587217737683.

Shyni, G.L., Kavitha, S., Indu, S., Arya, A.D., Anusree, S.S., Vineetha, V.P., et al. (2014). Chebulagic acid from Terminalia chebula enhances insulin mediated glucose uptake in 3T3-L1 adipocytes via PPARγ signaling pathway. *Biofactors* 40(6)**,** 646-657. doi: 10.1002/biof.1193.

Silawat, N., and Gupta, V.B. (2013). Chebulic acid attenuates ischemia reperfusion induced biochemical alteration in diabetic rats. *Pharm Biol* 51(1)**,** 23-29. doi: 10.3109/13880209.2012.698288.

Singh, I., Singh, P.K., Bhansali, S., Shafiq, N., Malhotra, S., Pandhi, P., et al. (2010). Effects of three different doses of a fruit extract of Terminalia chebula on metabolic components of metabolic syndrome, in a rat model. *Phytother Res* 24(1)**,** 107-112. doi: 10.1002/ptr.2879.

Singh, T.R., Gupta, L.N., and Kumar, N. (2016). Standard manufacturing procedure of Teekshna lauha bhasma. *J Ayurveda Integr Med* 7(2)**,** 100-108. doi: 10.1016/j.jaim.2015.08.003.

Sotoudeh, R., Hadjzadeh, M.A., Gholamnezhad, Z., and Aghaei, A. (2019). The anti-diabetic and antioxidant effects of a combination of Commiphora mukul, Commiphora myrrha and Terminalia chebula in diabetic rats. *Avicenna J Phytomed* 9(5)**,** 454-464.

Srinivasan, P., Vijayakumar, S., Kothandaraman, S., and Palani, M. (2018). Anti-diabetic activity of quercetin extracted from Phyllanthus emblica L. fruit: In silico and *in vivo* approaches. *J Pharm Anal* 8(2)**,** 109-118. doi: 10.1016/j.jpha.2017.10.005.

Sun, Y.M., Peng, L., and Li, Z.M. (2014). Determination of α-Glucosidase Inhibitory Activity of Huidouba Extracts by HPLC. *Nat Prod Res Dev* 26(10)**,** 1673-1676+1689. doi: 10.16333/j.1001-6880.2014.10.028.

Suo, N.C. (2019). The Clinical Value of Tibetan Medicine Shibawei Hezi Liniao Wan in the Treatment of Diabetes. *World Latest Med Inf* 19(56)**,** 227-228. doi: 10.19613/j.cnki.1671-3141.2019.56.139.

Suryanarayana, P., Kumar, P.A., Saraswat, M., Petrash, J.M., and Reddy, G.B. (2004). Inhibition of aldose reductase by tannoid principles of Emblica officinalis: implications for the prevention of sugar cataract. *Mol Vis* 10**,** 148-154.

Suryanarayana, P., Saraswat, M., Petrash, J.M., and Reddy, G.B. (2007). Emblica officinalis and its enriched tannoids delay streptozotocin-induced diabetic cataract in rats. *Mol Vis* 13**,** 1291-1297.

Suryavanshi, S.V., Garud, M.S., Barve, K., Addepalli, V., Utpat, S.V., and Kulkarni, Y.A. (2020). Triphala Ameliorates Nephropathy via Inhibition of TGF-β1 and Oxidative Stress in Diabetic Rats. *Pharmacology* 105(11-12)**,** 681-691. doi: 10.1159/000508238.

Tan, J., Wei, H.T., Zhang, Y.L., and Wang, Y.G. (2019). Regulating effect of Siwei-Jianghuangtang powder on PI3K/Akt signaling pathway in diabetic nephropathy rats. *Int J Tradit Chin Med* (04)**,** 387-393. doi: 10.3760/cma.j.issn.1673-4246.2019.04.015.

Tang, W.L., and Li, Y.H. (2021). Study on Active Ingredients of Tibetan Medicine Fumigated

Cattle for Lowering Blood Sugar. *Guangzhou Chem Ind* 49(09)**,** 81-83.

Taviad, K.K., Vekariya, S., Bedarkar, P., Galib, R., and Patgiri, B.J. (2018). Process standardization of Swarna Makshika Shodhana (purification) in Triphala Kwatha (decoction). *Ayu* 39(3)**,** 187-194. doi: 10.4103/ayu.AYU_26_18.

Tiwari, V., Kuhad, A., and Chopra, K. (2011). Emblica officinalis corrects functional, biochemical and molecular deficits in experimental diabetic neuropathy by targeting the oxido-nitrosative stress mediated inflammatory cascade. *Phytother Res* 25(10)**,** 1527-1536. doi: 10.1002/ptr.3440.

Tong, D., Wang, W.J., Luo, Y., Su, S.H., Zhou, K., Zhou, B.H., et al. (2018). Preliminary study of the dose-proportion relation of Tibetan medicine Siwei

Jianghuang Prescription on diabetic nephropathy rats induced by STZ. *Chin Tradit Pat Med* 40(03)**,** 516-524. doi: 10.3969/j.issn.1001-1528.2018.03.003.

Tuohetaerbaike, B., Zhang, Y., Tian, Y., Zhang, N.N., Kang, J., Mao, X., et al. (2020). Pancreas protective effects of Urolithin A on type 2 diabetic mice induced by high fat and streptozotocin via regulating autophagy and AKT/mTOR signaling pathway. *J Ethnopharmacol* 250**,** 112479. doi: 10.1016/j.jep.2019.112479.

Tupe, R.S., Kemse, N.G., Khaire, A.A., and Shaikh, S.A. (2017). Attenuation of glycation-induced multiple protein modifications by Indian antidiabetic plant extracts. *Pharm Biol* 55(1)**,** 68-75. doi: 10.1080/13880209.2016.1228683.

Tupe, R.S., Sankhe, N.M., Shaikh, S.A., Phatak, D.V., Parikh, J.U., Khaire, A.A., et al. (2015). Aqueous extract of some indigenous medicinal plants inhibits glycation at multiple stages and protects erythrocytes from oxidative damage-an *in vitro* study. *J Food Sci Technol* 52(4)**,** 1911-1923. doi: 10.1007/s13197-013-1211-8.

Upadya, H., Prabhu, S., Prasad, A., Subramanian, D., Gupta, S., and Goel, A. (2019). A randomized, double blind, placebo controlled, multicenter clinical trial to assess the efficacy and safety of Emblica officinalis extract in patients with dyslipidemia. *BMC Complement Altern Med* 19(1)**,** 27. doi: 10.1186/s12906-019-2430-y.

Usharani, P., Fatima, N., and Muralidhar, N. (2013). Effects of Phyllanthus emblica extract on endothelial dysfunction and biomarkers of oxidative stress in patients with type 2 diabetes mellitus: a randomized, double-blind, controlled study. *Diabetes Metab Syndr Obes* 6**,** 275-284. doi: 10.2147/dmso.S46341.

Variya, B.C., Bakrania, A.K., and Patel, S.S. (2020). Antidiabetic potential of gallic acid from Emblica officinalis: Improved glucose transporters and insulin sensitivity through PPAR-γ and Akt signaling. *Phytomedicine* 73**,** 152906. doi: 10.1016/j.phymed.2019.152906.

Wang, H., Shi, S., and Wang, S. (2018). Can highly cited herbs in ancient Traditional Chinese medicine formulas and modern publications predict therapeutic targets for diabetes mellitus? *J Ethnopharmacol* 213**,** 101-110. doi: 10.1016/j.jep.2017.10.032.

Wang, J.P., Xie, C.H., Jin, G., and Zhang, Y. (2015). Application of Turnip particles for diabetic retionopathy after retinal photocoagulation. *Chin J Ocul Traum Occupat Eye Dis* 37(05)**,** 372-375. doi: 10.3760/cma.j.issn.2095-1477.2015.05.014.

Wang, Q.Y., Xu, H.Y., Xu, Z.H., Lu, Z.M., Liu, M., and Shi, J.S. (2014). Hypoglycemic Effect of Water Extracts from *Edgeworthia gardneri* (Wall.) Meissn on Type 2 Diabetic Mice. *Nat Prod Res Dev* 26(9)**,** 1385-1388,1425. doi: 10.16333/j.1001-6880.2014.09.011.

Wang, Y.L., Xiao, Z.Q., Liu, S., Wan, L.S., Yue, Y.D., Zhang, Y.T., et al. (2013). Antidiabetic effects of Swertia macrosperma extracts in diabetic rats. *J Ethnopharmacol* 150(2)**,** 536-544. doi: 10.1016/j.jep.2013.08.053.

Wang, Y.X., Zhang, Z.W., REN, Y.L., Liu, M., Shi, J.S., Xu, Z.H., et al. (2019). The improvement and the mechanism of impaired islets in diabetes mellitus of *Edgeworthia gardneri* (Wall.) Meissn. *Nat Prod Res Dev* 31(3)**,** 506-511. doi: 10.16333/j.1001-6880.2019.3.022.

Wang, Z.C., Yang, L.X., Li, X.Y., Jiang, L.E., Zhang, B.N., Sun, W., et al. (2016). Effect of Total Flavonoids of *Oxytropis Falcate* Bunge on Blood Glucose and Insulin Resistance in Type 2 Diabetic KKAy Mice. *Chin J Exp Tradit Med Form* 22(22)**,** 113-117. doi: 10.13422/j.cnki.syfjx.2016220113.

Wei, R.N., Shao, M.S., and Wang, L. (2021). Exploration of clinical application and dosage of medicine Terminalia fruit. *J Changchun Univ Chin Med* 37(01)**,** 28-31. doi: 10.13463/j.cnki.cczyy.2021.01.008.

Wei, Y.F., Yang, Y.J., Li, J.Y., Duan, B.W., and Li, Y. (2011). Study on hypoglycemic effect of Tibetan medicine Liucha on alloxan-induced diabetic mice. *Lishizhen Med Mate Med Res* 22(10)**,** 2460-2461. doi: 10.3969/j.issn.1008-0805.2011.10.063.

Westfall, S., Lomis, N., and Prakash, S. (2018). A novel polyphenolic prebiotic and probiotic formulation have synergistic effects on the gut microbiota influencing Drosophila melanogaster physiology. *Artif Cells Nanomed Biotechnol* 46(sup2)**,** 441-455. doi: 10.1080/21691401.2018.1458731.

Wu, Y., Deng, W.R., and Wu, S.M. (2017). Protective effect and mechanism of *Oxytropis falcata* Bunge on myocardial ischemia reperfusion injury in diabetic rats. *Chin J Gerontol* 37(08)**,** 1884-1886.

Wu, Y., Wang, X.N., Chen, F.F., Chen, Y.N., and Tian, S.Q. (2013). Active ingredients of Huidouba in the treatment of type 2 diabetes mellitus. *J Chin Med Mater* 36(08)**,** 1313-1316. doi: 10.13863/j.issn1001-4454.2013.08.030.

Xie, J., Nie, Q.F., and Wu, X.L. (2018). Effects of *Lamiophlomis rotata* on glial cell activation in spinal dorsal horn of rats

with diabetic pain. *Chin Tradi Pat Med* 40(12)**,** 2624-2629. doi: 10.3969/j.issn.1001-1528.2018.12.003.

Xu, T., Ge, Y., Du, H., Li, Q., Xu, X., Yi, H., et al. (2021). Berberis kansuensis extract alleviates type 2 diabetes in rats by regulating gut microbiota composition. *J Ethnopharmacol* 273**,** 113995. doi: 10.1016/j.jep.2021.113995.

Xu, W., Li, J., Shao, R., Yan, X.H., and Wei, P. (2015). Extraction process of Huidouba-protein by ultrasound and its inhibition on α-glucosidase. *Sci Technol Food Ind* 36(14)**,** 254-259. doi: 10.13386/j.issn1002-0306.2015.14.044.

Yadav, S.S., Singh, M.K., Singh, P.K., and Kumar, V. (2017). Traditional knowledge to clinical trials: A review on therapeutic actions of Emblica officinalis. *Biomed Pharmacother* 93**,** 1292-1302. doi: 10.1016/j.biopha.2017.07.065.

Yan, C., Wang, Q.H., Wang, J.Y., Chang, B.Q., Huang, S., Li, B., et al. (2020). *Nepeta Angustifolia* Extract on Hypoglycemic Effect of Mice with Diabetes. *Chin Arch Tradit Chin Med* 38(01)**,** 140-143. doi: 10.13193/j.issn.1673-7717.2020.01.033.

Yang, K., Bai, Y., Yu, N., Lu, B., Han, G., Yin, C., et al. (2020). Huidouba Improved Podocyte Injury by Down-Regulating Nox4 Expression in Rats With Diabetic Nephropathy. *Front Pharmacol* 11**,** 587995. doi: 10.3389/fphar.2020.587995.

Yang, K.B., and Kang, Z.R. (2017). Research progress on treatment of diabetes with Tibetan medicine Huidouba *Chin Tradit Herb Drugs* 48(08)**,** 1682-1686. doi: 10.7501/j.issn.0253-2670.2017.08.031

Yang, L., Wang, Z., Jiang, L., Sun, W., Fan, Q., and Liu, T. (2017a). Total Flavonoids Extracted from *Oxytropis falcata* Bunge Improve Insulin Resistance through Regulation on the IKKβ/NF-κB Inflammatory Pathway. *Evid Based Complement Alternat Med* 2017**,** 2405124. doi: 10.1155/2017/2405124.

Yang, L.X., Fan, Q., Jiang, L.E., Meng, X.Y., Wang, Y.S., and Liu, T.H. (2017b). Effect of Total Flavonoids Extracted from *Oxytropis falcata* Bunge on Inflammatory Cytokines in Insulin Resistant 3T3-L1 Adipocytes. *Tradit Chin Med Res* 30(01)**,** 66-70.

Yang, L.X., Wang, Z.C., Li, X.Y., Zhang, B.N., Guo, M., Jiang, L.E., et al. (2016). Effect of Total Flavonoids Extracted from *Oxytropis falcata* Bunge on Blood Lipid Profile, Leptin, Adiponectin and Resistin. *Tradit Chin Med Res* 29(04)**,** 61-63.

Yang, M.H., Vasquez, Y., Ali, Z., Khan, I.A., and Khan, S.I. (2013). Constituents from Terminalia species increase PPARα and PPARγ levels and stimulate glucose uptake without enhancing adipocyte differentiation. *J Ethnopharmacol* 149(2)**,** 490-498. doi: 10.1016/j.jep.2013.07.003.

Yao, X.C., Chen, X.H., Duan, Y.B., and Li, X.Y. (2015). Effect of N-Butanol Extract of *Brassica rapa* on Blood Glucose Level of Alloxan-induced Diabetic Mice. *Nat Prod Ｒes Dev* 27(04)**,** 706-709+731. doi: 10.16333/j.1001-6880.2015.04.029.

Ye, F., Yue, L.J., Fan, G., Meng, X.L., and Lai, G.R. (2016). Effects of water extract from cortex of *Berberi dictyophylla* on diabetic retinopathy in spontaneous Type 2 diabetic *db/db* Mice (Ⅰ). *Chin J Exp Tradit Med Form* 22(02)**,** 82-86. doi: 10.13422/j.cnki.syfjx.2016020082.

Yue, L.J., Meng, X.L., Zhang, Y., Xiang, L., Fan, G., and Lai, X.R. (2014). Effect of Tibetan Medicine Berberis Cortex on Expressions of PKC-β, VEGF,

HIF-1α in Retina of Diabetic Rats. *Mod Tradit Chin Med Mater Med-World Sci Technol* (1)**,** 181-186. doi: 10.11842/wst.2014.01.034.

Yue, L.J., Zhang, Y., Xiang, L., Lai, X.R., and Meng, X.L. (2013). Study on the Effect of *Berberis dictyophlla* Cortex on Diabetic Retinopathy and the Mechanism. *Chin J Exp Tradit Med Form* 19(20)**,** 149-153. doi: 10.11653/syfj2013200149.

Zafar, T.A., Allafi, A.R., Alkandari, D., and Al-Othman, A. (2021). Rheological characteristics of wheat-chickpea composite flour doughs and effect of Amla powder (Phyllanthus emblica L.) addition on the functional properties of bread. *Food Sci Technol Int* 27(3)**,** 264-275. doi: 10.1177/1082013220950068.

Zhang, X.R., Qiao, Y.J., Zhu, H.T., Kong, Q.H., Wang, D., Yang, C.R., et al. (2021a). Multiple *in vitro* biological effects of phenolic compounds from Terminalia chebula var. tomentella. *J Ethnopharmacol* 275**,** 114135. doi: 10.1016/j.jep.2021.114135.

Zhang, Y., Meng, X.L., Yue, L.J., Xiang, L., and Lai, X.R. (2013). Preliminary Studies on Tibetan Medicine Berberis Cortex on blood Glucose

Level in Diabetic Mellitus Mice. *Prog Mod Biomed* 13(19)**,** 3619-3622,3607. doi: 10.13241/j.cnki.pmb.2013.19.011.

Zhang, Y., Xiang, R., Fang, S., Huang, K., Fan, Y., and Liu, T. (2020a). Experimental Study on the Effect of Tibetan Medicine Triphala on the Proliferation and Apoptosis of Pancreatic Islet β Cells through Incretin-cAMP Signaling Pathway. *Biol Pharm Bull* 43(2)**,** 289-295. doi: 10.1248/bpb.b19-00562.

Zhang, Y., Yan, L.S., Ding, Y., Cheng, B.C.Y., Luo, G., Kong, J., et al. (2020b). Edgeworthia gardneri (Wall.) Meisn. Water Extract Ameliorates Palmitate Induced Insulin Resistance by Regulating IRS1/GSK3β/FoxO1 Signaling Pathway in Human HepG2 Hepatocytes. *Front Pharmaco* 10**,** 1666. doi: 10.3389/fphar.2019.01666.

Zhang, Y., Zhang, Y., Halemahebai, G., Tian, L., Dong, H., and Aisker, G. (2021b). Urolithin A, a pomegranate metabolite, protects pancreatic β cells from apoptosis by activating autophagy. *J Ethnopharmacol* 272**,** 113628. doi: 10.1016/j.jep.2020.113628.

Zhang, Z.W., Xu, H.Y., Zhao, H., Geng, Y., Ren, Y.L., Guo, L., et al. (2019). *Edgeworthia gardneri* (Wall.) Meisn. water extract improves diabetes and modulates gut microbiota. *J Ethnopharmaco* 239**,** 111854. doi: 10.1016/j.jep.2019.111854.

Zhao, D.G., Zhou, A.Y., Du, Z.Y., Zhang, Y., Zhang, K., and Ma, Y.Y. (2015). Coumarins with α-glucosidase and α-amylase inhibitory activities from the flower of Edgeworthia gardneri. *Fitoterapia* 107**,** 122-127. doi: 10.1016/j.fitote.2015.10.012.

Zhao, J.H., and Tian, S.Q. (2008). Study on diabetes mellitus therapeutic effects of traditional Chinese medicine Compound HDB *J Southwest Minzu Univ* (*Nat Sci Ed*) 34(06)**,** 1186-1188.

Zhao, J.Q., Wang, Y.M., Yang, Y.L., Zeng, Y., Mei, L.J., Shi, Y.P., et al. (2017). Antioxidants and α-glucosidase inhibitors from "Liucha" (young leaves and shoots of Sibiraea laevigata). *Food Chem* 230**,** 117-124. doi: 10.1016/j.foodchem.2017.03.024.

Zhao, M., Song, J.J., Wang, Z.Z., Liu, W., and Liu, Y. (2011). Preventive and Therapeutic Effects of Compound Preparation of Huidouba on Diabetes. *Food and Drug* 13(07)**,** 254-257.

Zhao, Y., Feng, H., Zhou, Z., Hao, L., Xiang, Y.N., Wang, X.Y., et al. (2019). Ideas for New Drug Discovery of Prevention and Treatment of Diabetic Nephropathy Based on“Holistic View”in Tibetan Medicine. *Chin J Exp Tradit Med Form* 25(03)**,** 167-172. doi: 10.13422/j.cnki.syfjx.20182202.

Zheng, L.L., Wang, G., and Wu, Q. (2021). Effects of Eighteen Flavor *Myrobalan* Diuretic pills on the signaling pathway of RAGE/NF-κB in renal tissues of male rats with diabetic nephropathy. *Chin High Alt Med Biol* 42(02)**,** 120-125. doi: 10.13452/j.cnki.jqmc.2021.02.008.

Zhnag, F.B., Chen, X.H., Wang, S.L., Hu, C.H., Weng, Y.X., and Yang, S.B. (2017). The Effects of n-butyl Alcohol Extracted from Plateau Plant WuJing on the Related Biochemical Indexes of Diabetic Mice Models. *Western J Tradit Chin Med* 30(11)**,** 15-17.

Zhou, B.H., Ye, F., Yue, L.J., Meng, X.L., Fan, G., and Lai, X.R. (2016). Effects of water extract from cortex of *Berberi dictyophylla* on diabetic retinopathy in spontaneous Type 2 diabetic *db/db* Mice (Ⅱ). *Chin J Exp Tradit Med Form* 22(24)**,** 116-121. doi: 10.13422/j.cnki.syfjx.2016240116.

Zhou, J., Zhang, C., Zheng, G.H., and Qiu, Z. (2018). Emblic Leafflower (Phyllanthus emblica L.) Fruits Ameliorate Vascular Smooth Muscle Cell Dysfunction in Hyperglycemia: An Underlying Mechanism Involved in Ellagitannin Metabolite Urolithin A. *Evid Based Complement Alternat Med* 2018**,** 8478943. doi: 10.1155/2018/8478943.

Zhou, Y.L., Jia, X.B., Liu, J.P., and Feng, L. (2018). Protection of Huidouba, a Tibetan medicine on renal injury in STZ-induced type Ⅱ diabetic mice. *Chin Tradi Pat Med* 40(03)**,** 505-511. doi: 10.3969/j.issn.1001-1528.2018.03.001.

Zhuang, M., Qiu, H., Li, P., Hu, L., Wang, Y., and Rao, L. (2018). Islet protection and amelioration of type 2 diabetes mellitus by treatment with quercetin from the flowers of Edgeworthia gardneri. *Drug Des Devel Ther* 12**,** 955-966. doi: 10.2147/dddt.S153898.

Zuo, X.S., Ma, D.Q., Fang, S.D., Hua, J., Fan, Y., and Zhang, Y. (2018). Inhibition of High Glucose-induced Apoptosis of Pancreatic β-cells by Gallic Acid in *Phyllanthus Emblica*. *J Kunming Med Univ* 39(06)**,** 14-21.
